# Increasing nodal vulnerability and nodal efficiency implied recovery time prolonging in patients with supplementary motor area syndrome

**DOI:** 10.1002/hbm.25896

**Published:** 2022-05-04

**Authors:** Shengyu Fang, Lianwang Li, Shimeng Weng, Zhong Zhang, Xing Fan, Tao Jiang, Yinyan Wang

**Affiliations:** ^1^ Department of Neurosurgery, Beijing Tiantan Hospital Capital Medical University Beijing China; ^2^ Beijing Neurosurgical Institute Capital Medical University Beijing China; ^3^ Research Unit of Accurate Diagnosis, Treatment, and Translational Medicine of Brain Tumors Chinese Academy of Medical Sciences Beijing China

**Keywords:** awake craniotomy, causal mediation analysis, glioma, supplementary motor area syndrome, topological properties

## Abstract

Supplementary motor area (SMA) syndrome is a surgery‐related complication that commonly occurs after removing SMA glioma, and needs weeks to recover. However, susceptible factors of patients suffering from SMA syndrome remain unknown. Graphic theory was applied to reveal topological properties of sensorimotor network (SMN) by processing resting‐state functional magnetic resonance images in 66 patients with SMA gliomas. Patients were classified into SMA and non‐SMA groups based on whether they suffered from SMA syndrome. We collected recovery time and used causal mediation analysis to find association between topological properties and recovery time. Compared with the non‐SMA group, higher vulnerability (left: *p* = .0018; right: *p* = .0033) and lower fault tolerance (left: *p* = .0022; right: *p* = .0248) of the whole SMN were found in the SMA group. Moreover, higher nodal properties of lesional‐hemispheric cingulate cortex (nodal efficiency: left, *p* = .0389; right, *p* = .0169; nodal vulnerability: left, *p* = .0185; right, *p* = .0085) and upper limb region of primary motor cortex (PMC; nodal efficiency: left, *p* = .0132; right, *p* = .0001; nodal vulnerability: left, *p* = .0091; right, *p* = .0209) were found in the SMA group. Nodal efficiency and nodal vulnerability of cingulate cortex and upper limb region of PMC were important predictors for SMA syndrome occurring and recovery time prolonging. Neurosurgeons should carefully deal with upper limb region of PMC and cingulate cortex, and protect them if these two region were unnecessary to damage during SMA glioma resection.

## 1 INTRODUCTION

Supplementary motor area (SMA) syndrome is a specific surgery‐related condition that commonly occurs after the removal of a glioma from the SMA (Kim et al., 2013). The syndrome is characterized by global or partial akinesia within 24 hours after tumor resection, and with patients recovering in weeks to months (Nakajima et al., 2015). To date, SMA remains a blind area for intraoperative neurophysiological monitoring, although awaken craniotomy (AC) can provide a relative direct reflection of the functional state of SMA, the SMA syndrome was unable to completely avoid (Krainik et al., 2004; Rosenberg et al., 2010).

Resection of lesioned‐hemispheric primary motor cortex (PMC), cingulate gyrus are associated with SMA syndrome occurring (Fontaine et al., 2002; Kim et al., 2013). Smaller regions of the contralesional SMA being activated preoperatively (Krainik et al., 2004), resection of the cingulate cortex, and paralysis occurring 7 days postoperatively were negative factors for SMA syndrome recovery (Nakajima et al., 2019). Moreover, contralesional SMA plays an important role in replacing the lesioned‐hemispheric SMA to contribute to this syndrome recover through sensorimotor network (SMN) remodeling. (Desmurget et al., 2007; Oda et al., 2018).

Unfortunately, previous studies are not sufficient to identify who are susceptible for suffering from SMA syndrome (Potgieser et al., 2014). Nelson et al. (2002) found that a small activated region of the contralesional SMA in preoperative task functional magnetic resonance imaging (fMRI) might be related to patients with SMA syndrome (Russell & Kelly, 2003). However, these small activated regions might also be associated with increased transcallosal inhibition due to the glioma (Shimizu et al., 2002) and not related to SMA syndrome. Lacking of evidence, how the damage of lesioned PMC, cingulate cortex, even contralesional SMA cause SMA syndrome is not well understood.

Glioma is a progressive disease, and is able to induce neuroplasticity at surrounding tissues (Bulubas et al., 2020). Hence, as a part of SMN, when a glioma grew in the SMA, SMN remodeling plays a key role in SMA syndrome occurrence and recovery (Potgieser et al., 2014). Using resting‐state fMRI (rs‐fMRI), Vassal et al. (2017) found alterations in the functional connectivity (FC) of interhemispheric connections at different time points during recovery from SMA syndrome. However, how the association between SMN remodeling and patients suffering from SMA syndrome is unclear. In this study, we applied a graphic theoretical measurement and causal mediation analysis to identify what alterations in the SMN were susceptible for patients with SMA syndrome after AC.

## 2 METHODS

### 2.1 Patients

Eighty patients with low grade SMA gliomas who underwent AC between January 2016 and August 2020 at the Beijing Tiantan Hospital were reviewed retrospectively. The inclusion criteria were as follows: (1) adult patients; (2) no history of surgical treatment or radiotherapy; and (3) gliomas mainly focused on the SMA. The exclusion criteria were as follows: (1) glioma‐inducing midline shifting; and (2) head motion >1 mm in translation or 1° in rotation. After matching general information (e.g., sex, age, and education level), 33 healthy right handness subjects were included as controls in this study. All participants provided informed written consent prior to data acquisition. Institutional review board of Beijing Tiantan Hospital had approved this study.

### 2.2 Clinical characteristics

Pre‐ and postoperative Karnofsky Performance Scores (KPS), and histopathology was collected from inpatient recordings. The motor status was assessed by muscle strength test (UK Medical Research Council) before AC, on the second day of tumor resection, at 7 days, and 14 days postoperatively. Follow‐up information about the time of recovery of motor function (a maximum of 6 months after AC) was obtained by outpatient interviews. Patients were classified into the SMA and non‐SMA groups based on the presence of transient paralysis of the contralesional limbs at the second day of tumor resection. Recovery time was defined as the period from tumor resection to motor function recovery back to preoperative status. In addition, isocitrate dehydrogenase mutation (IDH) mutation status was assessed by pyrosequencing, and the type of IDH1 mutation was R132H.

### 2.3 Motor mapping procedure

AC was completed by one of the authors, who had more than 15 years of experience in performing the procedure. The procedure of motor mapping was the same as that reported in previous studies (Fang, Bai, et al., 2020; Fang et al., 2021). A brief summary was that we used bipolar stimulators to identify motor eloquent area according to the cortex or subcortical fibers with positive reactions and removed the areas of tumors without positive reactions.

### 2.4 MRI acquisition

A 3.0 T MR scanner of MAGNETOM Prisma (Siemens) was used to acquire image data of T2‐fluid‐attenuated inversion recovery (FLAIR), and rs‐fMRI images before surgery within 72 hours. Regarding the resting state fMRI scan, all patients were asked to keep awaking and did not thinking anything. The scan time of rs‐fMRI was 8 min. Postoperative T2‐FLAIR images were acquired within 48 hours and at the 7 days after tumor resection. Parameter information of MRI sequences is shown in Part 1 of Supporting Information Materials S1.

### 2.5 Regions of tumor invasion

One of the authors manually drew tumor masks based on the hyperintension of T2‐FLAIR images, and another author with 10 years of experience made the final decision about tumor masks. All masks were normalized to the MNI‐152 T2 template with SPM 8 software (University College London; http://www.fil.ion.ucl.ac.uk/spm/). Tumor volume was calculated using the volumetric method using MRIcron software (http://www.mccauslandcenter.sc.edu/mricro/mricron/) based on the tumor masks (Figure S1).

### 2.6 Rs‐fMRI data preprocessing

A brief summary was that we used the theoretical network analysis software (https://www.nitrc.org/projects/gretna) to preprocess rs‐fMRI data with the sequential steps of transform data, removing initial images, slice timing, realignment, normalization, smoothing, temporal detrending, regressing out covariance, temporal filtering, and scrubbing (detailed information in Supporting Information Materials S1).

### 2.7 Regions of interest

To avoid the errors of spatial normalization induced by tumor occupation, we first excluded regions that were invaded by the glioma, and then extracted remaining parts of SMN from a brain atlas “brant 274” (http://www.brainnetome.org/) (Fan et al., 2016). Due to the cingulate cortex was related to SMA syndrome occurring, we added the bilateral cingulate cortex into the calculating template. All seeds were generated as 5‐mm spheres based on the coordinates in the template of “brant 274” (Tables S1 and S2).

### 2.8 Matrix of FC construction

To construct a matrix of FC, the mean time series between the each two nodes of the calculating template were analyzed with Pearson's correlation. All FC matrices were processed by Z transformation.

### 2.9 Graph theory measures

Graph theory measurement was used to generate global and nodal properties. Global properties include clustering coefficient, global efficiency, shortest path length, local efficiency, transitivity, vulnerability, and fault tolerance. Nodal properties include efficiency, local efficiency, degree centrality, and vulnerability. The positive and weighted FC matrices were used to calculate topological properties with a series of sparsity (0.10–0.40, interval 0.01) (Laney et al., 2015).

### 2.10 Statistical analyses

Clinical data were compared between the patient and healthy groups using Student's *t*‐test, chi‐square test, and one‐way analysis of variance (ANOVA) based on the type of data. Bonferroni correction was used to correct FC results. Topological properties were compared among the groups using one‐way ANOVA. Depending on whether the variance was homogeneous, the Tamhane or Bonferroni correction was subsequently used for post hoc analysis.

**FIGURE 1:**
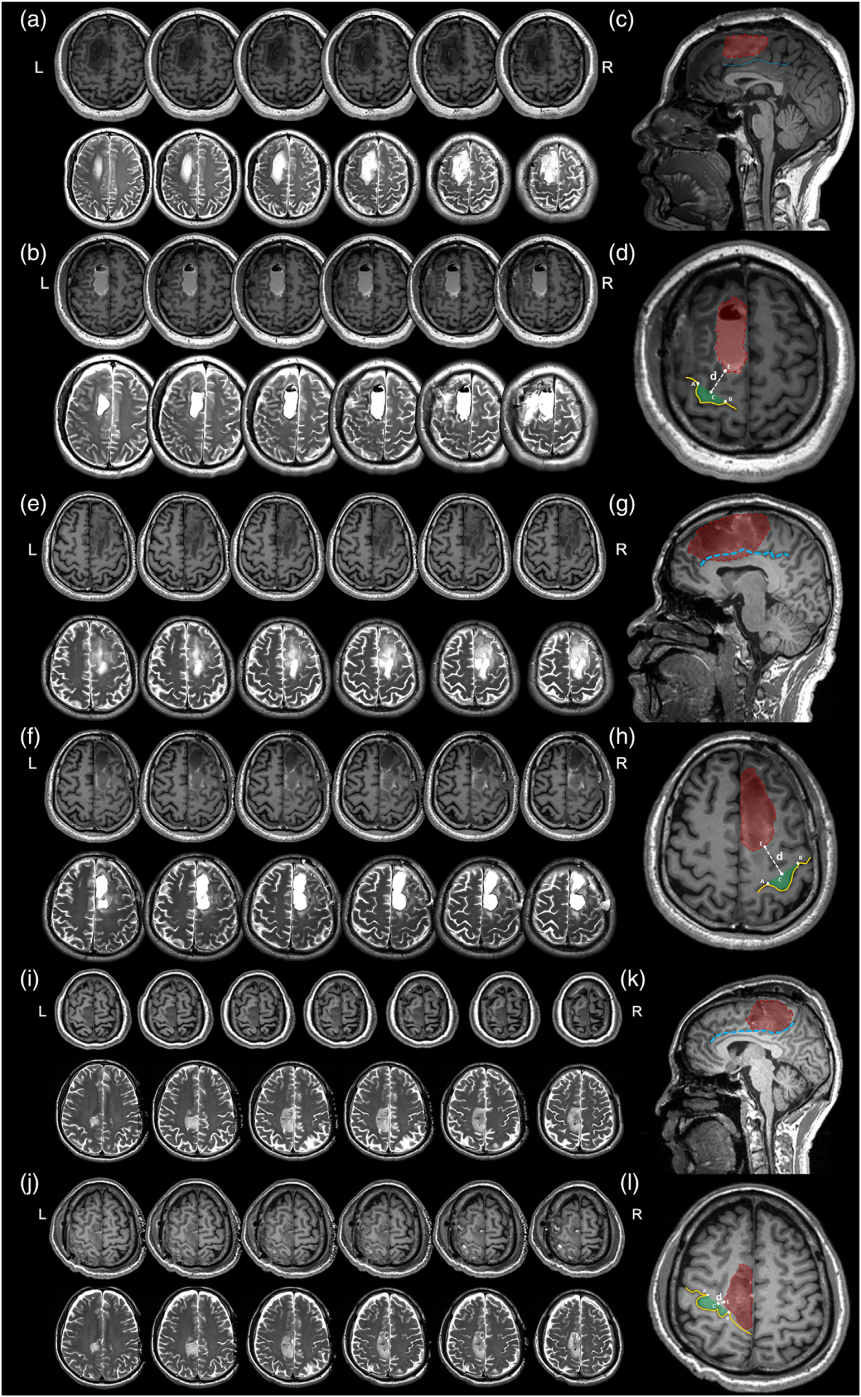
The locations of tumor, upper limb region of BA 4 (A4ul), and cingulate cortex. (a–d) Glioma did not involve in cingulate cortex or A4ul. (e–h) Glioma did not involve in A4ul but involve in cingulate cortex. (i–l) Glioma did not involve in cingulate cortex but involve in A4ul. (a, e, and i) Preoperative T1 and T2 images; (b, f, and j). Postoperative T1 and T2 images. (c, g, and k) Extent of tumor resection and location of cingulate cortex. The blue dotted line was cingulate sulcus. The red region was extent of tumor resection. (d, h, and l) Extent of tumor resection and location of the A4ul. The red region was extent of tumor resection, and the green region was the hand knob that represents the A4ul to region of tumor resection. If the *d* < 5 mm, the extent of tumor resection was defined as adjacent to/involving in the A4ul. Point C was the midpoint of Point A and Point B that two endpoints of hand knob. Point E was the point which was nearest to the Point C.

Pearson's correlation analysis and causal mediation analysis were used to find the factors of SMA syndrome recovery by SPSS software (v19.0; IBM) and package of PROCESS v3.0 (Preacher & Hayes, 2004). In the procedure of causal mediation analysis (model number = 4; confidence interval = 95; and number of bootstrap = 5000), independent variable was whether region of tumor resection adjacent to/involved in PMC or cingulate cortex (Figure 1). Mediator were nodal properties of PMC or cingulate cortex, and the dependent variable was recovery time of patients in the SMA group regardless of whether the glioma was in the left or right hemisphere.

### 2.11 Data availability

Anonymized data will be made available on request.

## 3 RESULTS

**FIGURE 2:**
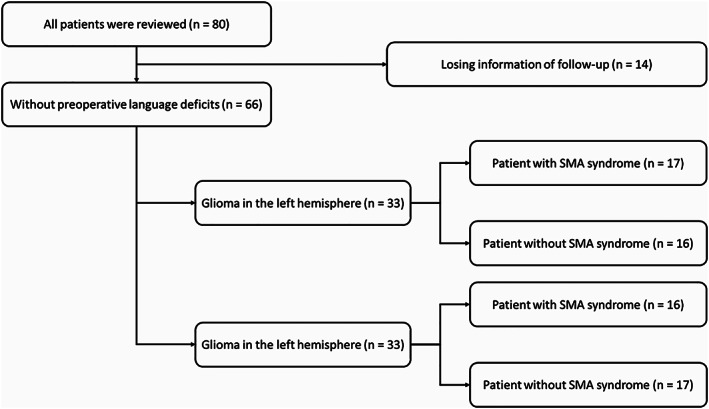
The information of patient recruitment

All of 66 patients were included in the study (14 excluded because they lost follow‐up information, Figure 2), with 33 in the SMA group (17 with tumors in the left hemisphere) and 33 in the non‐SMA group (16 with tumors in the left hemisphere). All patients were right‐handed, and all tumors were completely resected.

No differences in KPS, ratio of histopathology, IDH, chromosome 1p/19q codeletion, or tumor volume were observed between the SMA and non‐SMA groups or between tumors growing in the left or right hemisphere (Tables 1 and 2).

**TABLE 1:** Clinical characteristics (glioma grew in left hemisphere)

Demographic and clinical characteristics	SMA group (*n* = 17)	Non‐SMA group (*n* = 16)	Healthy (*n* = 33)	*p* Value
Gender				.67
Male	10	7	18	
Female	7	9	15	
Age (years)^a^	43.6 ± 2.3	38.3 ± 2.4	37.2 ± 1.5	.49
Handness				
Right	17	16	33	–
Left	0	0	0	
KPS score (preoperative)				
100	17	16	33	>.99
90	0	0	0	
80	0	0	0	
Education period (years)^a^	12.1 ± 0.6	12.6 ± 0.8	13.4 ± 0.6	.99
IDH status			–	.85
Mutation	9	9		
Wild‐type	8	7		
Chromosome 1p/19q status			–	.62
Codeletion	5	6		
Non‐codeletion	12	10		
MGMT premotor methylation				
Methylation	10	9		.88
Non‐methylation	7	7		
TERT promoter mutation				.38
Mutation	13	10		
Wild‐type	4	6		
Tumor volume (ml)^a^	28.64 ± 3.61	28.88 ± 3.72	–	.96

*Note*: Using Student *t*‐test to compare the difference of tumor volume and KPS between the SMA and non‐SMA groups. Using one‐way ANOVA test to compare the differences of age and education period between the SMA and non‐SMA, and healthy groups. Using to chi‐square test to compare the differences of gender, tumor grade, IDH status, chromosome 1p/19q status, MGMT premotor methylation, and TERT promotor mutation between the SMA and non‐SMA groups.

Abbreviations: KPS, Karnofsky Performance Score; MGMT, O^6^‐methylguanine DNA methyltransferase; non‐SMA group, group of patients without supplementary motor area syndrome; SMA group, group of patients with supplementary motor area syndrome; TERT, telomerase reverse transcriptase gene.

^a^ Values are means ± standard error of mean.

**TABLE 2:** Clinical characteristics (glioma grew in right hemisphere)

Demographic and clinical characteristics	SMA group (*n* = 16)	Non‐SMA group (*n* = 17)	Healthy (*n* = 33)	*p* Value
Gender				.61
Male	9	7	18	
Female	7	10	15	
Age (years)^a^	44.2 ± 1.9	41.1 ± 3.2	37.2 ± 1.5	.49
Handness				
Right	16	17	33	–
Left	0	0	0	
KPS score (preoperative)				
100	16	17	33	>.99
90	0	0	0	
80	0	0	0	
Education period (years)^a^	12.1 ± 0.8	13.4 ± 0.8	13.4 ± 0.6	.99
IDH status			–	.62
Mutation	9	11		
Wild‐type	7	6		
Chromosome 1p/19q status			–	.58
Codeletion	6	8		
Non‐codeletion	10	9		
MGMT premotor methylation			–	.55
Methylation	11	10		
Non‐methylation	5	7		
TERT promotor mutation			–	.62
Mutation	10	12		
Wild‐type	6	5		
Tumor volume (ml)^a^	23.41 ± 3.79	33.18 ± 5.14	–	.14

*Note*: Using Student *t*‐test to compare the difference of tumor volume and KPS between the SMA and non‐SMA groups. Using one‐way ANOVA test to compare the differences of age and education period between the SMA and non‐SMA, and healthy groups. Using to chi‐square test to compare the differences of gender, tumor grade, IDH status, chromosome 1p/19q status, MGMT premotor methylation, and TERT promotor mutation between the SMA and non‐SMA groups.

Abbreviations: KPS, Karnofsky Performance Score; MGMT, O^6^‐methylguanine DNA methyltransferase; non‐SMA group, group of patients without supplementary motor area syndrome; SMA group, group of patients with supplementary motor area syndrome; TERT, telomerase reverse transcriptase gene.

^a^ Values are means ± standard error of mean.

### 3.1 FC differences

Compared with the non‐SMA group, only one edge that connected the node of contralesional medial Brodmann area (BA) 6 to the node of lesioned‐hemispheric BA 2 significantly decreased FC in the SMA group after bonferroni correction (*p* < .0001, Table S3). A large number of edges in the SMN have shown significantly decreased FC in the SMA and non‐SMA groups compared with the healthy group after Bonferroni correction.

### 3.2 Differences in global topological properties

**FIGURE 3:**
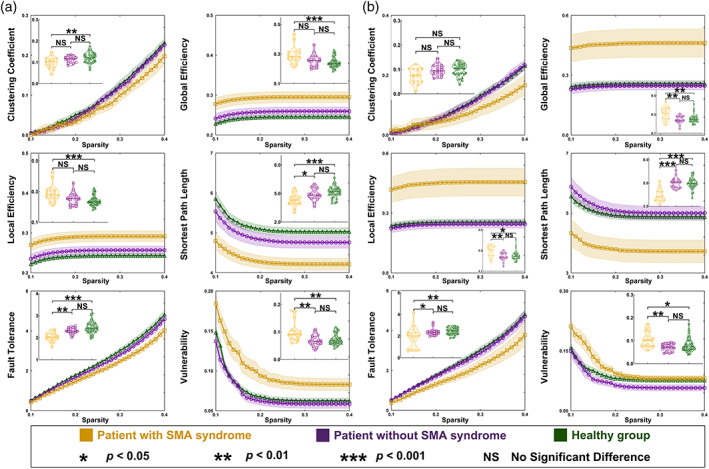
Differences of global properties between the SMA, non‐SMA, and healthy groups. (a) Comparison results when the glioma was in the left hemisphere. (b) Comparison results when the glioma was in the right hemisphere. BA, Brodmann area; SMA, supplementary motor area

The results of global topological properties obtained by one‐way ANOVA showed that whether the glioma was in the left or right hemisphere, global efficiency, local efficiency, shortest path length, fault tolerance, and vulnerability were significantly different between the three groups (the SMA, non‐SMA, and healthy groups). The results after post hoc analysis with Bonferroni correction were shown as follows:

Regarding the left hemispheric glioma (Table S4 and Figure 3a), the shortest path length (*p* = .0351) and fault tolerance (*p* = .0022) were weaker in the SMA group than those in the non‐SMA group. Vulnerability in the SMA group was greater than that in the non‐SMA group (*p* = .0018).

Regarding the right hemispheric glioma (Table S5 and Figure 3b), the shortest path length (*p* < .0001) and fault tolerance (*p* = .0248) were weaker in the SMA group than those in the non‐SMA group. Global efficiency (*p* = .0024), local efficiency (*p* = .0064), vulnerability (*p* = .0033) in the SMA group were greater than those in the non‐SMA group.

### 3.3 Differences in nodal properties

**FIGURE 4:**
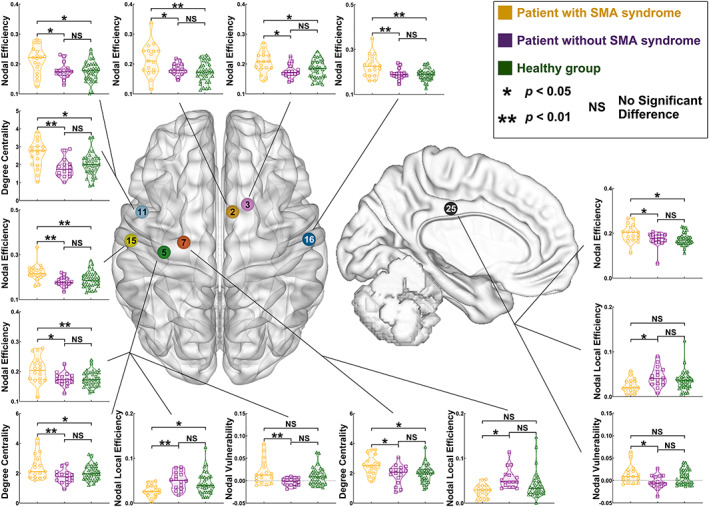
Differences of nodal properties between the SMA, non‐SMA, and healthy groups when the glioma was in the left hemisphere. Node no. 2 (dark yellow) = medial BA 6; node no. 3 (pink) = ventrolateral BA 6; node no. 5 (green) = upper limb of BA 4; node no. 7 (green) = trunk region of BA 4; node no. 11 (light blue) = caudal ventrolateral BA 6; node no. 15 (light yellow) = left hemispheric upper limb and face of BA 1/2/3; node no. 16 (dark blue) = right hemispheric upper limb and face of BA 1/2/3; node 25 (black) = caudal area BA 23 (cingulate cortex). BA, Brodmann area; SMA, supplementary motor area

Regarding the left hemispheric glioma (Figure 4), the results of nodal properties after post hoc analysis with Bonferroni correction were shown as follows:

Compared with the non‐SMA group, the SMA group increased nodal efficiency (Table S6) in the right hemispheric nodes that were medial BA 6 (A6m, *p* = .0460), ventrolateral BA 6 (A6vl, *p* = .0195), upper limb and face of BA 1/2/3 (A1_2_3ulhf, *p* = .0072), and the left hemispheric nodes that were upper limb of BA 4 (A4ul, *p* = .0132), caudal ventrolateral BA 6 (A6cvl, *p* = .0127), A1_2_3ulhf (*p* = .0019), caudal area BA 23 (A23c, *p* = .0389). Moreover, the SMA group decreased nodal local efficiency (Table S7) in the left hemispheric nodes that were A4ul (*p* = .0078), trunk region of BA 4 (A4t, *p* = .0066), and A23c (*p* = .0258) compared with the non‐SMA group. In addition, the SMA group increased nodal degree of centrality (Table S8) in the left hemispheric nodes that were A4ul (*p* = .0028), A4t (*p* = .0153), and A6cvl (*p* = .0037) compared with the non‐SMA group. Furthermore, the SMA group increased nodal vulnerability (Table S9) in the left hemispheric nodes that were A4ul (*p* = .0091), and A23c (*p* = .0185) compared with the non‐SMA group.

**FIGURE 5:**
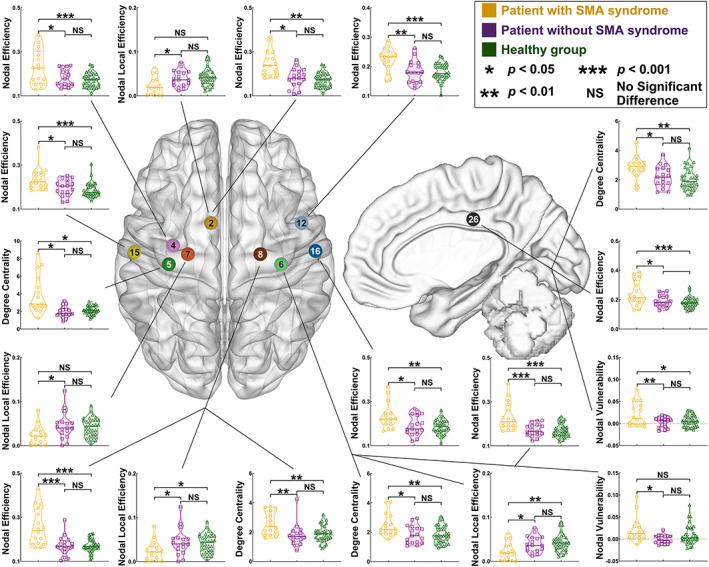
Differences of nodal properties between the SMA, non‐SMA, and healthy groups when the glioma was in the left hemisphere. Node no. 2 (dark yellow) = medial BA 6; node no. 4 (pink) = caudal dorsolateral BA 6; node no. 5 (dark green) = left hemispheric upper limb of BA 4; node no. 6 (light green) = right hemispheric upper limb of BA 4node; node no. 7 (orange) = trunk region of BA 4; node no. 8 (brown) = trunk region of BA 4; node no. 12 (light blue) = caudal ventrolateral BA 6; node no. 15 (light yellow) = left hemispheric upper limb and face of BA 1/2/3; node no. 16 (dark blue) = right hemispheric upper limb and face of BA 1/2/3; node 26 (black) = caudal area BA 23 (cingulate cortex). BA, Brodmann area; SMA, supplementary motor area

Regarding the right hemispheric glioma (Figure 5), the results of nodal properties after post hoc analysis with bonferroni or Tamhane correction were shown as follows:

Compared with the non‐SMA group, the SMA group increased nodal efficiency (Table S10) in the left hemispheric nodes that were A6m (*p* = .0245), caudal dorsolateral BA 6 (A6cdl, *p* = .0172), A1_2_3ulhf, (*p* = .0157), and the right hemispheric nodes that were A4ul, (*p* = .0001), A4t (*p* < .0001), A6cvl (*p* = .0019), A1_2_3ulhf (*p* = .0145), A23c (*p* = .0169). Moreover, the SMA group decreased nodal local efficiency (Table S11) in the left hemispheric nodes that were A6m (*p* = .0237), A4t (*p* = .0299), caudal‐dorsal BA 24 (A24cd, *p* = .0406), A23c (*p* = .0387), and right hemispheric nodes that were A4ul (*p* = .0384), A4t (*p* = .0413), trunk region of BA 1/2/3 (*p* = .0116), A24cd (*p* = .0181) compared with the non‐SMA group. In addition, the SMA group increased nodal degree of centrality (Table S12) in the left hemispheric node that was A4ul (*p* = .0319) and right hemispheric nodes that were, A4ul (*p* = .0150), A4t (*p* = .0029), and A23c (*p* = .0462) compared with the non‐SMA group. Furthermore, the SMA group increased nodal vulnerability (Table S13) in the right hemispheric nodes that were A4ul (*p* = .0209), and A23c (*p* = .0085) compared with the non‐SMA group.

### 3.4 Correlation analysis results

The results of correlation analysis indicated that nodal efficiency of the nodes A4ul and A23c were positively correlated with recovery time (A4ul, *r* = .594, *p* = .0003; A23c, *r* = .541, *p* = .0011), respectively. Moreover, nodal vulnerability of the nodes A4ul and A23c were positively correlated with recovery time (A4ul, *r* = .518, *p* = .0020; A23c, *r* = .544, *p* = .0011), respectively.

### 3.5 Causal mediation results

**FIGURE 6:**
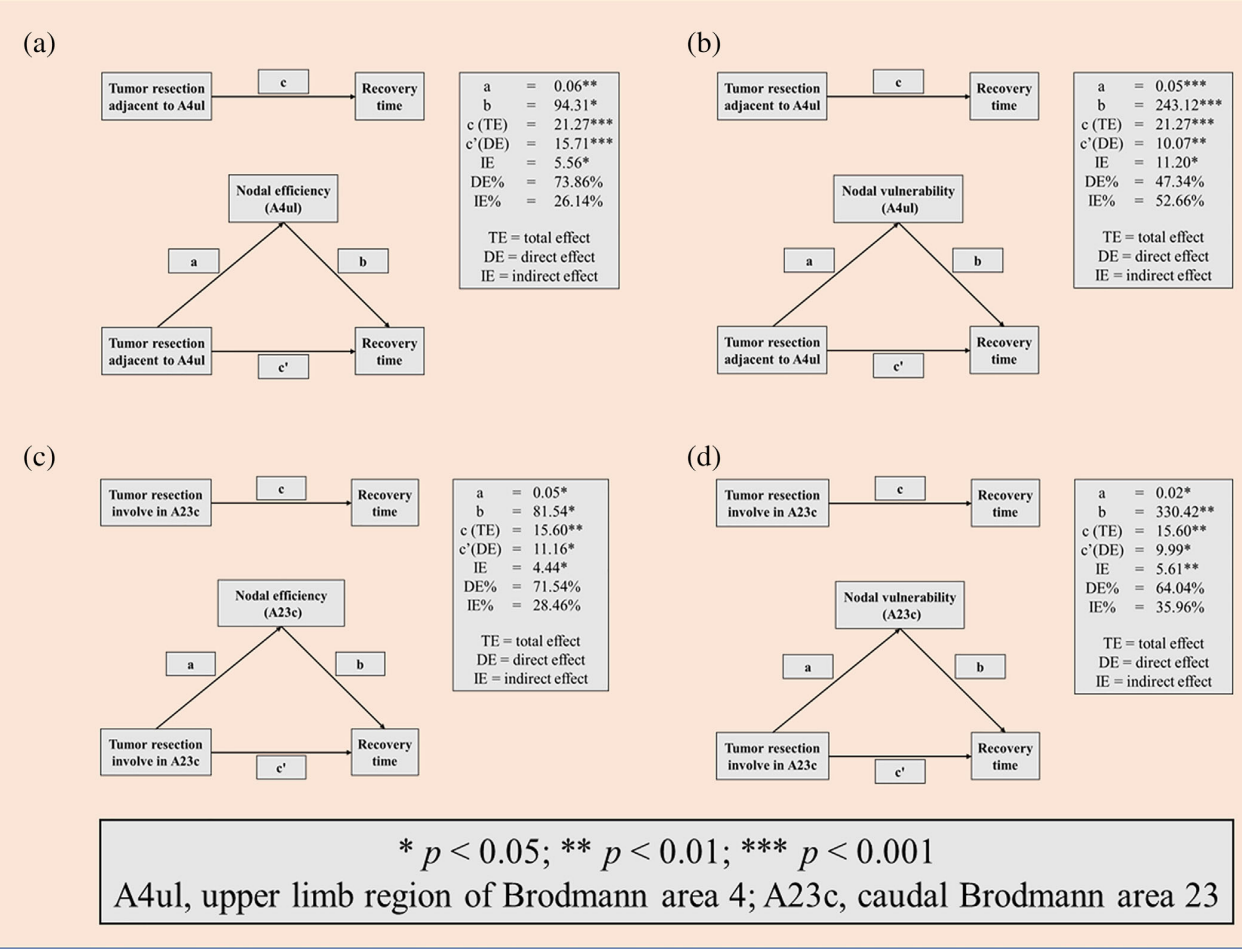
Causal mediation analysis of recovery time of SMA syndrome and nodal topological properties. The main factor to causing SMA syndrome and prolonged recovery time was that nodes (A4ul and A23c) were affected by surgical regions and postoperative edema, and increased nodal efficiency and nodal vulnerability before surgery of these two nodes mediated this phenomenon; SMA, supplementary motor area

The results of causal mediation analysis (Figure 6 and Tables S14–S17) showed that nodal efficiency of the node A4ul was a mediated factor between surgical region or edema area involved in A4ul and recovery time of motor functions in postoperation (total effect = 21.27, direct effect [DE] = 15.71, indirect effect [IE] = 5.56, DE% = 73.86%, IE% = 26.14%). Similarly, nodal vulnerability of the node A4ul was mediated factor (total effect = 21.27, DE = 10.07, IE = 11.20, DE% = 47.34%, IE% = 52.66%). Moreover, nodal efficiency (total effect = 15.6, DE = 11.16, IE = 4.44, DE% = 71.54%, IE% = 28.46%) and nodal vulnerability (total effect = 15.6, DE = 9.99, IE = 5.61, DE% = 64.04%, IE% = 35.96%) of the node A23c were mediated factors between whether surgical region or edema involved in A23c and recovery time of motor functions in postoperation.

## 4 DISCUSSION

Based on the preoperative rs‐fMRI images, this study explored the differences in FC and topological properties of the SMN between patients with or without SMA syndrome after AC treatment. We found that SMA syndrome occurring is associated with higher vulnerability and lower fault tolerance of the whole SMN, and higher nodal efficiency and nodal vulnerability of the nodes (A23c, cingulate area, and A4ul, region of upper limb on BA 4).

A previous study showed that more than 90% of the extent of SMA resection was related to SMA syndrome (Russell & Kelly, 2003). However, in our study, all patients underwent total resection of SMA glioma, and while some developed SMA syndrome, others did not. This pointed toward other causes for SMA syndrome, which might be related to SMN plasticity. Because low grade glioma with slow growth contributes to network reorganize and compensate for the damaged functions (Potgieser et al., 2014). Our FC results, a lot of connections decreased their FC value in patients groups compared with the healthy group, indicated that the glioma growing in the SMA indeed altered SMN. The SMA was an important cortex to control motor function (Rosenberg et al., 2010), and no patients with preoperative motor deficits might indicate that the SMN reorganized and compensated for the damage motor function.

The role of contralesional SMA is controversial in terms of predicting whether SMA syndrome occurs (Nelson et al., 2002; Potgieser et al., 2014). Although some studies have shown that more activated regions of the contralesional SMA are helpful for the recovery of SMA syndrome (Krainik et al., 2004), they are not sufficient to avoid SMA syndrome (Rosenberg et al., 2010). A previous study showed that contralesional SMA was important for SMA syndrome recovering through strengthening the connections between ipsilesional primary sensory area and contralesional SMA (Vassal et al., 2017). However, that study (Vassal et al., 2017) only enrolled six patients and flipped images of patients to make the number of data was able to perform statistical analysis. Hence, our FC result further verified that contralesional SMA was important for SMA syndrome occurring, which was the connection from the contralesional SMA to the ipsilesional primary sensory area significantly decreased FC in the SMA group compared with that in the non‐SMA group.

The shortest path length reflects the lowest cost for all nodes to transmit information to other nodes in the whole network (Fang, Zhou, et al., 2020). Lower cost of information conveying, the higher ability of information conveying the network has. Compared with the non‐SMA group and healthy group, the decreased shortest path length in the SMA group indicated that the ability of information conveying was strengthened after the glioma appeared. We thought that increased ability of information conveying was related to network reorganization that was induced by glioma as previous discussion (Potgieser et al., 2014). Moreover, nodal efficiency of a node means the count backwards of the shortest path length of the subnetwork that this node participated in, and means the ability of information conveying of this node (Fox & King, 2018). Similar findings indicated that the PMC (A4ul) and cingulate cortex (A23c) were reorganized, which nodal efficiency of PMC (A4ul) and cingulate cortex (A23c) was increased in the SMA group. However, why did these patients with the stronger ability of information conveying in the SMN suffer from SMA syndrome? We thought that it was related to the reimpairment of reorganized network that induced by tumor resection.

Vulnerability and fault tolerance reflects strike‐resistance in a network (Latora & Marchiori, 2005). Vulnerability is a ratio that altered global efficiency divided global efficiency of original network when nodes were impaired and lost their ability compared with the original network (Latora & Marchiori, 2005). Moreover, the fault tolerance reflects the maximum number of nodes that were impaired and lost their ability but did not change the global efficiency of the network (Tchernev et al., 2005). Hence, the higher vulnerability and lower fault tolerance indicated the network was less stable. Previous findings indicated that gliomas induced network unstable (Fang et al., 2021; Kinno et al., 2014). Our results found similar alterations in patients with SMA syndrome (higher vulnerability and lower fault tolerance in the SMA group). With the higher vulnerability and lower fault tolerance, the ability of conveying will reduce more obviously when the network is impaired. The SMA glioma resection is the reason that leads to the SMN network be impaired.

Moreover, nodal efficiency represents the ability of information conveying througha a node. Nodal vulnerability represents a ratio of global efficiency altering when a node was impaired and lost their ability in subnetwork that this node participant in. A previous study found that if the cingulate cortex was damaged during SMA tumor resection, the patients would spend over 1 month to recovery from SMA syndrome (Nakajima et al., 2019). Our results of correlation analysis showed that if the preoperative nodal efficiency and nodal vulnerability of the nodes A4ul and A23c were high, the recovery time was unexpected long. What caused this phenomenon? The surgical region adjacent to PMC or involved in cingulate cortex indicated that the tumor was closed to these two regions. Hence, when tumor resection caused these two nodes disruption, the rest of SMN would be affected obviously in patients with high nodal efficiency and nodal vulnerability compared with patients with a low nodal efficiency and nodal vulnerability. Thus, it indicated that the lesioned‐hemispheric cingulate cortex and upper limb region of PMC were important nodes for SMA syndrome occurring (Kuang et al., 2020; Vassal et al., 2017; Wang et al., 2021).

In addition, to further investigate the determined factor of prolonging recovery time, causal mediation analysis was applied (Preacher & Hayes, 2004). Based on our results, we confirmed that the surgical region adjacent to the PMC of upper limb and cingulate cortex were the major factors to prolong recovery time of SMA syndrome compared with nodal efficiency of A4ul and A23c and nodal vulnerability of A23c. Since the proportion of IEs of those nodal properties in the causal mediation analysis were lower than 50%. However, nodal vulnerability of A4ul was the most influenced factor for prolonging recovery time compared with the affection of tumor resection because the proportion of IE was higher than 50%. Hence, neurosurgeons should carefully deal with upper limb region of PMC and cingulate cortex, and protect them if these two region were unnecessary to damage during SMA glioma resection. If one of these two regions were damaged during SMA glioma resection, it predicts that patients need far longer to recovery their motor functions. Furthermore, due to the PMC of upper limb was an important node that nodal efficiency and nodal vulnerability of this node were related to prolonging recovery time, and the PMC of upper limb was stimulated more easily than cingulate cortex (in a deep location) by neuro‐navigated transcranial repetitive magnetic stimulation, this point might become a stimulated target to facilitate motor function recovery.

## 5 CONCLUSION

The glioma grew on the SMA and induced SMN remodeling. The ability of information conveying was strengthened through increasing nodal efficiency and sacrificing stability of SMN in some patients. Lesional‐hemispheric upper limb region of PMC and cingulate cortex were two important nodes, and nodal efficiency and nodal vulnerability were two important properties for foreseeing SMA syndrome occurring and recovering especially for patients with one of these two nodes impairment during SMA glioma resection. Neurosurgeons should carefully deal with upper limb region of PMC and cingulate cortex, and protect them if these two region were unnecessary to damage during SMA glioma resection.

## AUTHOR CONTRIBUTIONS

*Study concept and design*: Shengyu Fang, Lianwang Li, and Shimeng Weng. *Data acquisition and analysis*: Shengyu Fang, Shimeng Weng, and Xing Fan. *Statistics/verified analytical method*: Shengyu Fang, Lianwang Li, and Yinyan Wang. *Writing the first draft*: Shengyu Fang and Yinyan Wang. *Supervision study*: Xing Fan, Yinyan Wang, and Tao Jiang. *Read and approved final version*: All authors.

## Supporting information


**Appendix S1** Supporting Information

## Data Availability

Anonymized data will be made available on request.
