# Supplementary material for: Increasing nodal vulnerability and nodal efficiency implied recovery time prolonging in patients with supplementary motor area syndrome
Source: Hum Brain Mapp. 2022 May 4;43(13):3958–69. doi: 10.1002/hbm.25896 (PMC9374886; doi:10.1002/hbm.25896)
Supplement: Supplementary file 1 — Appendix S1 Supporting Information [file HBM-43-3958-s001.docx]

# Title: Impairment of cingulate cortex or precentral gyrus is an underlying cause of prolonging recovery time of motor deficits

# Supplementary materials

# Part 1. Parameters of MRI sequences

All MR images were acquired using a MAGNETOM Prisma 3T MR scanner (Siemens, Erlangen, Germany) within 72 hours before tumor resection.

T2-FLAIR sequence were used to collect glioma images. The parameters were as follows: repetition time (TR): 5000 ms; echo time (TE): 105 ms; flip angle: 150°; field of view (FOV): 240 mm × 240 mm; voxel size: 0.5 mm × 0.5 mm × 3 mm; slice number: 33.

Echo-planar imaging sequence was used for collecting resting state functional MRI. The parameters were as follows: TR: 2000 ms; TE: 30 ms; flip angle: 75°; FOV: 220 mm × 220 mm; voxel size: 3.0 mm × 3.0 mm × 5.0 mm; slice number: 30; acquisition duration: 8 minutes. All subjects were request to open their eyes, not think anything, and looked at the monitor which showed a black background and white cross during scanning.

# Part 2. Supplemental Tables

**Table S1. Montreal Neurological Institute (MNI) locations of 30 nodes in the sensorimotor network for left hemispheric glioma**

| **Regions of interesting** | **Modified Cyto-architectonic** | **Left hemisphere** | | | **Right hemisphere** | | |
| --- | --- | --- | --- | --- | --- | --- | --- |
|  |  | X | Y | Z | X | Y | Z |
| A6dl_R | Dorsolateral BA 6 | - | - | - | 19.7 | 4.3 | 64.3 |
| A6m_R | Medial BA 6 | - | - | - | 7.5 | -4.5 | 60.0 |
| A6vl_R | Ventrolateral BA 6 | - | - | - | 34.5 | 7.5 | 54.0 |
| A6cdl_R | Caudal dorsolateral BA 6 | - | - | - | 33.0 | -7.5 | 57.0 |
| A4ul_L(R) | Area BA 4 (upper limb) | -25.5 | -25.5 | 63.0 | 34.3 | -19.3 | 58.7 |
| A4t_L(R) | Area BA 4 (trunk) | -13.3 | -19.7 | 73.3 | 15.0 | -22.5 | 70.5 |
| A4tl_L(R) | Area BA 4 (tongue and larynx) | -52.5 | 0.0 | 7.5 | 54.0 | 4.5 | 9.0 |
| A6cvl_L(R) | Caudal ventrolateral BA 6 | -49.5 | 4.5 | 30.0 | 51.0 | 7.5 | 30.0 |
| A1_2_3ll_R | Area BA 1/2/3 (lower limb) | - | - | - | 10.5 | -34.5 | 54.0 |
| A4ll_R | Area BA 4 (lower limb) | - | - | - | 4.5 | -21.0 | 61.5 |
| A1_2_3ulhf_L(R) | Area BA 1/2/3 (upper limb and face) | -49.7 | -16.3 | 43.3 | 49.7 | -13.7 | 43.7 |
| A1_2_3tonIa_L(R) | Area BA 1/2/3 (tongue and larynx) | -55.7 | -13.7 | 16.3 | 55.5 | -10.5 | 15.0 |
| A2_L(R) | Area BA 2 | -46.5 | -30.0 | 49.5 | 48.0 | -24.0 | 48.0 |
| A1_2_3tru_L(R) | Area BA 1/2/3 (trunk) | -21.0 | -34.5 | 67.5 | 19.5 | -33.0 | 69.0 |
| A24cd_L(R) | Caudodorsal area BA 24 | -4.7 | 7.3 | 37.3 | 4.5 | 6.0 | 37.5 |
| A23c_L(R) | Caudal area BA 23 | -7.3 | -22.7 | 40.7 | 6.0 | -19.5 | 40.5 |
| mPMtha_L(R) | Pre-motor thalamus | -18.0 | -13.5 | 3.0 | 12.0 | -13.5 | 1.5 |
| Stha_L(R) | Sensory thalamus | -18.0 | -22.0 | 4.0 | 18.0 | -22.5 | 3.0 |

*****BA = Brodmann area.

**Table S2. Montreal Neurological Institute (MNI) locations of 30 nodes in the sensorimotor network for right hemispheric glioma**

| **Regions of interesting** | **Modified Cyto-architectonic** | **Left hemisphere** | | | **Right hemisphere** | | |
| --- | --- | --- | --- | --- | --- | --- | --- |
|  |  | X | Y | Z | X | Y | Z |
| A6dl_L | Dorsolateral BA 6 | -18.0 | -1.5 | 64.5 | - | - | - |
| A6m_ L | Medial BA 6 | -6.0 | -4.5 | 58.5 | - | - | - |
| A6vl_ L | Ventrolateral BA 6 | -31.7 | 4.3 | 55.3 | - | - | - |
| A6cdl_ L | Caudal dorsolateral BA 6 | -31.5 | -9.0 | 58.5 | - | - | - |
| A4ul_L(R) | Area BA 4 (upper limb) | -25.5 | -25.5 | 63.0 | 34.3 | -19.3 | 58.7 |
| A4t_L(R) | Area BA 4 (trunk) | -13.3 | -19.7 | 73.3 | 15.0 | -22.5 | 70.5 |
| A4tl_L(R) | Area BA 4 (tongue and larynx) | -52.5 | 0.0 | 7.5 | 54.0 | 4.5 | 9.0 |
| A6cvl_L(R) | Caudal ventrolateral BA 6 | -49.5 | 4.5 | 30.0 | 51.0 | 7.5 | 30.0 |
| A1_2_3ll_ L | Area BA 1/2/3 (lower limb) | -7.7 | -37.7 | 58.3 | - | - | - |
| A4ll_ L | Area BA 4 (lower limb) | -4.3 | -22.7 | 61.3 | - | - | - |
| A1_2_3ulhf_L(R) | Area BA 1/2/3 (upper limb and face) | -49.7 | -16.3 | 43.3 | 49.7 | -13.7 | 43.7 |
| A1_2_3tonIa_L(R) | Area BA 1/2/3 (tongue and larynx) | -55.7 | -13.7 | 16.3 | 55.5 | -10.5 | 15.0 |
| A2_L(R) | Area BA 2 | -46.5 | -30.0 | 49.5 | 48.0 | -24.0 | 48.0 |
| A1_2_3tru_L(R) | Area BA 1/2/3 (trunk) | -21.0 | -34.5 | 67.5 | 19.5 | -33.0 | 69.0 |
| A24cd_L(R) | Caudodorsal area BA 24 | -4.7 | 7.3 | 37.3 | 4.5 | 6.0 | 37.5 |
| A23c_L(R) | Caudal area BA 23 | -7.3 | -22.7 | 40.7 | 6.0 | -19.5 | 40.5 |
| mPMtha_L(R) | Pre-motor thalamus | -18.0 | -13.5 | 3.0 | 12.0 | -13.5 | 1.5 |
| Stha_L(R) | Sensory thalamus | -18.0 | -22.0 | 4.0 | 18.0 | -22.5 | 3.0 |

*****BA = Brodmann area.

**Table S3. Functional connectivity with significant difference between the SMA and non-SMA groups.**

| Functional Connectivity | Glioma in the left hemisphere | | | Glioma in the right hemisphere | | |
| --- | --- | --- | --- | --- | --- | --- |
|  | SMA group | non-SMA group | *p* value | SMA group | non-SMA group | *p* value |
| A6m_H and A2_L | -0.116 ± 0.040 | 0.121 ± 0.030 | 4.7×10^-5^ | -0.242 ± 0.077 | 0.175 ± 0.049 | 0.0001 |

***** SMA group = patients with supplementary motor area syndrome. Non-SMA group = patients without supplementary motor area syndrome. A6m_H = healthy hemispheric medial Brodmann Area 6; A2_L = lesional hemispheric Brodmann Area 2.

**Table S4. Global Topological properties compared between the patient and healthy groups when glioma grew in the left hemisphere**

|  | SMA group | non-SMA group | Health group | One-way ANOVA  (*p* value) | SMA vs non-SMA  (*p* value) | SMA vs Health  (*p* value) | non-SMA vs Health  (*p* value) |
| --- | --- | --- | --- | --- | --- | --- | --- |
| Global efficiency | 0.293 ± 0.014 | 0.257 ± 0.011 | 0.243 ± 0.006 | 0.0013 | 0.0614 | 0.0009 | 0.9027 |
| Local efficiency | 0.289 ± 0.014 | 0.254 ± 0.010 | 0.240 ± 0.006 | 0.0012 | 0.0590 | 0.0008 | 0.8771 |
| Shortest path length | 4.307 ± 0.163 | 4.877 ± 0.160 | 5.146 ± 0.105 | 0.0002 | 0.0351 | 0.0001 | 0.5012 |
| Clustering coefficient | 0.098 ± 0.006 | 0.114 ± 0.004 | 0.122 ± 0.004 | 0.0036 | 0.1899 | 0.0025 | 0.6217 |
| Vulnerability | 0.100 ± 0.008 | 0.068 ± 0.005 | 0.073 ± 0.003 | 0.0007 | 0.0018 | 0.0020 | > 0.9999 |
| Fault tolerance | 2.030 ± 0.051 | 2.308 ± 0.035 | 2.462 ± 0.046 | < 0.0001 | 0.0022 | < 0.0001 | 0.0863 |
| Transitivity | 0.026 ± 0.002 | 0.029 ± 0.001 | 0.030 ± 0.001 | 0.1478 | - | - | - |

***** The global properties were calculated with one-way ANOVA test. The value in the table = mean value ± standard error mean. Post-hoc analysis with bonferroni correction was applied. SMA group = patients with supplementary motor area syndrome. Non-SMA group = patients without supplementary motor area syndrome.

**Table S5. Global Topological properties compared between the patient and healthy groups when glioma grew in the right hemisphere**

|  | SMA group | non-SMA group | Health group | One-way ANOVA  (*p* value) | SMA vs non-SMA  (*p* value) | SMA vs Health  (*p* value) | non-SMA vs Health  (*p* value) |
| --- | --- | --- | --- | --- | --- | --- | --- |
| Global efficiency | 0.307 ± 0.017 | 0.245 ± 0.010 | 0.256 ± 0.008 | 0.0013 | 0.0024 | 0.0046 | > 0.9999 |
| Local efficiency^#^ | 0.256 ± 0.008 | 0.242 ± 0.010 | 0.252 ± 0.008 | 0.0044 | 0.0064 | 0.0148 | > 0.9999 |
| Shortest path length | 3.422 ± 0.206 | 5.021 ± 0.165 | 4.973 ± 0.125 | < 0.0001 | < 0.0001 | < 0.0001 | > 0.9999 |
| Clustering coefficient | 0.073 ± 0.023 | 0.097 ± 0.005 | 0.099 ± 0.004 | 0.2185 | - | - | - |
| Vulnerability^#^ | 0.105 ± 0.009 | 0.071 ± 0.004 | 0.082 ± 0.005 | 0.0037 | 0.0033 | 0.0332 | 0.5756 |
| Fault tolerance | 1.847 ± 0.245 | 2.373 ± 0.081 | 2.458 ± 0.054 | 0.0021 | 0.0248 | 0.0018 | > 0.9999 |
| Transitivity | 0.016 ± 0.004 | 0.023 ± 0.001 | 0.025 ± 0.001 | 0.0194 | 0.1163 | 0.0178 | > 0.9999 |

***** The global properties were calculated with one-way ANOVA test. The value in the table = mean value ± standard error mean. Post-hoc analysis with bonferroni correction was applied. SMA group = patients with supplementary motor area syndrome. Non-SMA group = patients without supplementary motor area syndrome.

**Table S6. Nodal efficiency compared between the patients and healthy groups** **when glioma grew in the left hemisphere**

| Node name | SMA group | non-SMA group | Health group | One-way ANOVA  (*p* value) | SMA vs non-SMA  (*p* value) | SMA vs Health  (*p* value) | non-SMA vs Health  (*p* value) |
| --- | --- | --- | --- | --- | --- | --- | --- |
| A6dl_R | 0.200 ± 0.008 | 0.169 ± 0.007 | 0.175 ± 0.006 | 0.0108 | 0.0191 | 0.0286 | > 0.9999 |
| A6m_R | 0.214 ± 0.013 | 0.182 ± 0.005 | 0.172 ± 0.006 | 0.0015 | 0.0460 | 0.0011 | > 0.9999 |
| A6vl_R | 0.205 ± 0.008 | 0.176 ± 0.006 | 0.182 ± 0.005 | 0.0128 | 0.0195 | 0.0369 | > 0.9999 |
| A6cdl_R | 0.201 ± 0.010 | 0.177 ± 0.009 | 0.168 ± 0.006 | 0.0181 | 0.2522 | 0.0145 | > 0.9999 |
| A4ul_L | 0.205 ± 0.011 | 0.174 ± 0.006 | 0.175 ± 0.004 | 0.0032 | 0.0132 | 0.0049 | > 0.9999 |
| A4ul_R | 0.212 ± 0.011 | 0.182 ± 0.005 | 0.175 ± 0.005 | 0.0017 | 0.0413 | 0.0013 | > 0.9999 |
| A4t_L | 0.216 ± 0.007 | 0.176 ± 0.009 | 0.181 ± 0.005 | 0.0002 | 0.0009 | 0.0006 | > 0.9999 |
| A4t_R | 0.196 ± 0.011 | 0.189 ± 0.007 | 0.174 ± 0.005 | 0.0779 | - | - | - |
| A4tl_L | 0.187 ± 0.010 | 0.172 ± 0.005 | 0.168 ± 0.005 | 0.1209 | - | - | - |
| A4tl_R | 0.190 ± 0.009 | 0.179 ± 0.006 | 0.159 ± 0.005 | 0.0034 | 0.9094 | 0.0037 | 0.1303 |
| A6cvl_L | 0.213 ± 0.011 | 0.177 ± 0.007 | 0.176 ± 0.006 | 0.0020 | 0.0127 | 0.0127 | > 0.9999 |
| A6cvl_R | 0.195 ± 0.014 | 0.185 ± 0.005 | 0.177 ± 0.005 | 0.2616 | - | - | - |
| A1_2_3ll_ R | 0.203 ± 0.006 | 0.181 ± 0.007 | 0.170 ± 0.005 | 0.0006 | 0.0700 | 0.0004 | 0.6081 |
| A4ll_ R | 0.207 ± 0.008 | 0.185 ± 0.007 | 0.176 ± 0.004 | 0.0014 | 0.0728 | 0.0010 | 0.9258 |
| A1_2_3ulhf_L | 0.221 ± 0.009 | 0.180 ± 0.005 | 0.189 ± 0.006 | 0.0011 | 0.0019 | 0.0056 | > 0.9999 |
| A1_2_3ulhf_R | 0.226 ± 0.012 | 0.190 ± 0.006 | 0.188 ± 0.004 | 0.0006 | 0.0072 | 0.0007 | > 0.9999 |
| A1_2_3tonIa_L | 0.196 ± 0.010 | 0.188 ± 0.007 | 0.174 ± 0.005 | 0.0807 | - | - | - |
| A1_2_3tonIa_R | 0.195 ± 0.011 | 0.185 ± 0.008 | 0.178 ± 0.005 | 0.2778 | - | - | - |
| A2_L | 0.204 ± 0.007 | 0.176 ± 0.005 | 0.182 ± 0.005 | 0.0079 | 0.0149 | 0.0207 | > 0.9999 |
| A2_R | 0.215 ± 0.010 | 0.183 ± 0.006 | 0.176 ± 0.006 | 0.0011 | 0.0297 | 0.0008 | > 0.9999 |
| A1_2_3tru_L | 0.218 ± 0.010 | 0.189 ± 0.007 | 0.178 ± 0.006 | 0.0015 | 0.0780 | 0.0010 | 0.9041 |
| A1_2_3tru_R | 0.204 ± 0.011 | 0.187 ± 0.005 | 0.175 ± 0.005 | 0.0120 | 0.4053 | 0.0094 | 0.6765 |
| A24cd_L | 0.207 ± 0.008 | 0.192 ± 0.007 | 0.188 ± 0.005 | 0.1334 | - | - | - |
| A24cd_R | 0.212 ± 0.009 | 0.194 ± 0.005 | 0.188 ± 0.005 | 0.0594 | - | - | - |
| A23c_L | 0.201 ± 0.010 | 0.172 ± 0.008 | 0.173 ± 0.005 | 0.0107 | 0.0389 | 0.0142 | > 0.9999 |
| A23c_R | 0.200 ± 0.006 | 0.184 ± 0.006 | 0.179 ± 0.005 | 0.0336 | 0.2719 | 0.0296 | > 0.9999 |
| mPMtha_L | 0.183 ± 0.006 | 0.165 ± 0.005 | 0.157 ± 0.005 | 0.0040 | 0.2719 | 0.0296 | > 0.9999 |
| mPMtha_R | 0.201 ± 0.010 | 0.186 ± 0.007 | 0.172 ± 0.005 | 0.0154 | 0.6509 | 0.0134 | 0.4894 |
| Stha_L | 0.179 ± 0.009 | 0.164 ± 0.008 | 0.161 ± 0.004 | 0.1379 | - | - | - |
| Stha_R | 0.189 ± 0.007 | 0.171 ± 0.006 | 0.165 ± 0.005 | 0.0228 | 0.2569 | 0.0189 | > 0.9999 |

***** The nodal efficiency was calculated with one-way ANOVA test. The value in the table = mean value ± standard error mean. Post-hoc analysis with bonferroni correction was applied. SMA group = patients with supplementary motor area syndrome. Non-SMA group = patients without supplementary motor area syndrome. **Table S7. Nodal local efficiency compared between the patients and healthy groups** **when glioma grew in the left hemisphere**

| Node name | SMA group | non-SMA group | Health group | One-way ANOVA  (*p* value) | SMA vs non-SMA  (*p* value) | SMA vs Health  (*p* value) | non-SMA vs Health  (*p* value) |
| --- | --- | --- | --- | --- | --- | --- | --- |
| A6dl_R | 0.035 ± 0.005 | 0.054 ± 0.009 | 0.040 ± 0.004 | 0.0914 | - | - | - |
| A6m_R | 0.032 ± 0.004 | 0.043 ± 0.003 | 0.042 ± 0.004 | 0.1839 | - | - | - |
| A6vl_R | 0.030 ± 0.004 | 0.036 ± 0.004 | 0.039 ± 0.005 | 0.4482 | - | - | - |
| A6cdl_R | 0.046 ± 0.008 | 0.042 ± 0.003 | 0.043 ± 0.005 | 0.8653 | - | - | - |
| A4ul_L | 0.028 ± 0.003 | 0.050 ± 0.005 | 0.044 ± 0.004 | 0.0060 | 0.0078 | 0.0270 | > 0.9999 |
| A4ul_R | 0.038 ± 0.005 | 0.048 ± 0.007 | 0.040 ± 0.004 | 0.4670 | - | - | - |
| A4t_L | 0.027 ± 0.004 | 0.055 ± 0.006 | 0.041 ± 0.005 | 0.0089 | 0.0066 | 0.2365 | 0.1857 |
| A4t_R | 0.036 ± 0.005 | 0.041 ± 0.004 | 0.041 ± 0.004 | 0.6701 | - | - | - |
| A4tl_L | 0.037 ± 0.005 | 0.039 ± 0.005 | 0.041 ± 0.004 | 0.7616 | - | - | - |
| A4tl_R | 0.038 ± 0.005 | 0.034 ± 0.006 | 0.048 ± 0.004 | 0.0907 | - | - | - |
| A6cvl_L | 0.034 ± 0.003 | 0.035 ± 0.005 | 0.034 ± 0.003 | 0.9884 | - | - | - |
| A6cvl_R | 0.032 ± 0.004 | 0.034 ± 0.006 | 0.045 ± 0.004 | 0.0876 | - | - | - |
| A1_2_3ll_ R | 0.038 ± 0.005 | 0.040 ± 0.005 | 0.035 ± 0.004 | 0.7218 | - | - | - |
| A4ll_ R | 0.042 ± 0.005 | 0.044 ± 0.004 | 0.044 ± 0.004 | 0.9799 | - | - | - |
| A1_2_3ulhf_L | 0.033 ± 0.004 | 0.043 ± 0.003 | 0.042 ± 0.004 | 0.2250 | - | - | - |
| A1_2_3ulhf_R | 0.038 ± 0.006 | 0.043 ± 0.003 | 0.037 ± 0.004 | 0.6760 | - | - | - |
| A1_2_3tonIa_L | 0.039 ± 0.004 | 0.037 ± 0.006 | 0.043 ± 0.004 | 0.7110 | - | - | - |
| A1_2_3tonIa_R | 0.038 ± 0.012 | 0.041 ± 0.006 | 0.038 ± 0.003 | 0.9284 | - | - | - |
| A2_L | 0.038 ± 0.008 | 0.043 ± 0.005 | 0.037 ± 0.003 | 0.6933 | - | - | - |
| A2_R | 0.042 ± 0.006 | 0.055 ± 0.007 | 0.041 ± 0.004 | 0.1963 | - | - | - |
| A1_2_3tru_L | 0.044 ± 0.006 | 0.037 ± 0.005 | 0.042 ± 0.004 | 0.7229 | - | - | - |
| A1_2_3tru_R | 0.037 ± 0.005 | 0.043 ± 0.005 | 0.041 ± 0.004 | 0.7661 | - | - | - |
| A24cd_L | 0.032 ± 0.006 | 0.039 ± 0.003 | 0.037 ± 0.003 | 0.6012 | - | - | - |
| A24cd_R | 0.043 ± 0.007 | 0.036 ± 0.005 | 0.041 ± 0.003 | 0.6571 | - | - | - |
| A23c_L | 0.025 ± 0.004 | 0.044 ± 0.005 | 0.036 ± 0.004 | 0.0293 | 0.0258 | 0.2213 | 0.5761 |
| A23c_R | 0.035 ± 0.005 | 0.041 ± 0.003 | 0.038 ± 0.003 | 0.6557 | - | - | - |
| mPMtha_L | 0.025 ± 0.004 | 0.034 ± 0.004 | 0.036 ± 0.004 | 0.1871 | - | - | - |
| mPMtha_R | 0.041 ± 0.006 | 0.039 ± 0.005 | 0.041 ± 0.003 | 0.9138 | - | - | - |
| Stha_L | 0.044 ± 0.009 | 0.036 ± 0.005 | 0.034 ± 0.004 | 0.5119 | - | - | - |
| Stha_R | 0.032 ± 0.004 | 0.041 ± 0.006 | 0.031 ± 0.003 | 0.2529 | - | - | - |

***** Nodal local efficiency was calculated with one-way ANOVA test. The value in the table = mean value ± standard error mean. Post-hoc analysis with bonferroni correction was applied. SMA group = patients with supplementary motor area syndrome. Non-SMA group = patients without supplementary motor area syndrome. **Table S8. Degree centrality compared between the patients and healthy groups** **when glioma grew in the left hemisphere**

| Node name | SMA group | non-SMA group | Health group | One-way ANOVA  (*p* value) | SMA vs non-SMA  (*p* value) | SMA vs Health  (*p* value) | non-SMA vs Health  (*p* value) |
| --- | --- | --- | --- | --- | --- | --- | --- |
| A6dl_R | 2.216 ± 0.139 | 1.787 ± 0.134 | 1.920 ± 0.114 | 0.1335 | - | - | - |
| A6m_R | 2.609 ± 0.261 | 2.108 ± 0.100 | 1.871 ± 0.108 | 0.0066 | 0.1903 | 0.0048 | > 0.9999 |
| A6vl_R | 2.261 ± 0.158 | 1.835 ± 0.107 | 2.061 ± 0.102 | 0.1312 | - | - | - |
| A6cdl_R | 2.205 ± 0.184 | 2.000 ± 0.214 | 1.819 ± 0.118 | 0.2395 | - | - | - |
| A4ul_L | 2.474 ± 0.207 | 1.753 ± 0.110 | 2.035 ± 0.086 | 0.0034 | 0.0028 | 0.0490 | 0.3753 |
| A4ul_R | 2.494 ± 0.248 | 1.965 ± 0.139 | 1.940 ± 0.092 | 0.0315 | 0.1228 | 0.0358 | > 0.9999 |
| A4t_L | 2.536 ± 0.157 | 1.923 ± 0.158 | 1.984 ± 0.100 | 0.0055 | 0.0153 | 0.0102 | > 0.9999 |
| A4t_R | 2.208 ± 0.205 | 2.157 ± 0.133 | 1.993 ± 0.094 | 0.4843 | - | - | - |
| A4tl_L | 1.937 ± 0.205 | 1.725 ± 0.086 | 1.842 ± 0.094 | 0.6276 | - | - | - |
| A4tl_R | 1.942 ± 0.145 | 1.979 ± 0.119 | 1.619 ± 0.087 | 0.0427 | > 0.9999 | 0.1416 | 0.1025 |
| A6cvl_L | 2.563 ± 0.182 | 1.810 ± 0.127 | 2.006 ± 0.110 | 0.0028 | 0.0037 | 0.0145 | 0.9514 |
| A6cvl_R | 2.243 ± 0.259 | 2.085 ± 0.140 | 1.963 ± 0.097 | 0.4524 | - | - | - |
| A1_2_3ll_ R | 2.308 ± 0.122 | 1.973 ± 0.142 | 1.831 ± 0.082 | 0.0115 | 0.2126 | 0.0088 | > 0.9999 |
| A4ll_ R | 2.521 ± 0.186 | 2.207 ± 0.178 | 2.037 ± 0.098 | 0.0631 | - | - | - |
| A1_2_3ulhf_L | 2.534 ± 0.182 | 2.073 ± 0.128 | 2.253 ± 0.105 | 0.1257 | - | - | - |
| A1_2_3ulhf_R | 2.721 ± 0.255 | 2.296 ± 0.168 | 2.144 ± 0.092 | 0.0415 | 0.3418 | 0.0367 | > 0.9999 |
| A1_2_3tonIa_L | 2.268 ± 0.229 | 2.228 ± 0.166 | 1.900 ± 0.094 | 0.1477 | - | - | - |
| A1_2_3tonIa_R | 2.182 ± 0.230 | 2.019 ± 0.161 | 2.086 ± 0.103 | 0.8159 | - | - | - |
| A2_L | 2.305 ± 0.173 | 1.936 ± 0.105 | 2.108 ± 0.084 | 0.1759 | - | - | - |
| A2_R | 2.537 ± 0.228 | 2.088 ± 0.115 | 1.981 ± 0.105 | 0.0323 | 0.2237 | 0.0294 | > 0.9999 |
| A1_2_3tru_L | 2.646 ± 0.220 | 2.195 ± 0.159 | 2.057 ± 0.118 | 0.0379 | 0.2908 | 0.0338 | > 0.9999 |
| A1_2_3tru_R | 2.291 ± 0.198 | 2.148 ± 0.105 | 1.995 ± 0.083 | 0.2388 | - | - | - |
| A24cd_L | 2.253 ± 0.215 | 2.269 ± 0.161 | 2.186 ± 0.101 | 0.9123 | - | - | - |
| A24cd_R | 2.680 ± 0.216 | 2.301 ± 0.099 | 2.218 ± 0.128 | 0.1127 | - | - | - |
| A23c_L | 2.121 ± 0.211 | 2.073 ± 0.091 | 1.945 ± 0.090 | 0.5930 | - | - | - |
| A23c_R | 2.255 ± 0.129 | 2.094 ± 0.135 | 2.019 ± 0.109 | 0.4218 | - | - | - |
| mPMtha_L | 1.829 ± 0.140 | 1.662 ± 0.097 | 1.538 ± 0.084 | 0.1572 | - | - | - |
| mPMtha_R | 2.254 ± 0.179 | 2.285 ± 0.168 | 2.003 ± 0.087 | 0.2366 | - | - | - |
| Stha_L | 1.801 ± 0.204 | 1.722 ± 0.127 | 1.675 ± 0.085 | 0.7924 | - | - | - |
| Stha_R | 2.038 ± 0.123 | 1.803 ± 0.135 | 1.723 ± 0.102 | 0.1864 | - | - | - |

***** The degree centrality was calculated with one-way ANOVA test. The value in the table = mean value ± standard error mean. Post-hoc analysis with bonferroni correction was applied. SMA group = patients with supplementary motor area syndrome. Non-SMA group = patients without supplementary motor area syndrome.

**Table S9. Nodal vulnerability compared between the patients and healthy groups** **when glioma grew in the left hemisphere**

| Node name | SMA group | non-SMA group | Health group | One-way ANOVA  (*p* value) | SMA vs non-SMA  (*p* value) | SMA vs Health  (*p* value) | non-SMA vs Health  (*p* value) |
| --- | --- | --- | --- | --- | --- | --- | --- |
| A6dl_R | 0.008 ± 0.004 | -0.001 ± 0.003 | 0.007 ± 0.003 | 0.2694 |  |  |  |
| A6m_R | 0.024 ± 0.009 | 0.011 ± 0.004 | 0.009 ± 0.004 | 0.1816 |  |  |  |
| A6vl_R | 0.013 ± 0.007 | 0.004 ± 0.003 | 0.009 ± 0.003 | 0.4664 |  |  |  |
| A6cdl_R | 0.009 ± 0.006 | 0.012 ± 0.006 | 0.011 ± 0.004 | 0.9383 |  |  |  |
| A4ul_L | 0.019 ± 0.007 | -0.002 ± 0.002 | 0.010 ± 0.003 | 0.0113 | 0.0091 | 0.4393 | 0.1230 |
| A4ul_R | 0.013 ± 0.006 | 0.007 ± 0.004 | 0.011 ± 0.004 | 0.6415 |  |  |  |
| A4t_L | 0.010 ± 0.003 | 0.003 ± 0.004 | 0.016 ± 0.004 | 0.1120 |  |  |  |
| A4t_R | 0.007 ± 0.005 | 0.008 ± 0.004 | 0.007 ± 0.003 | 0.9460 |  |  |  |
| A4tl_L | -0.004 ± 0.002 | -0.001 ± 0.002 | 0.007 ± 0.003 | 0.0343 | > 0.9999 | 0.0413 | 0.3335 |
| A4tl_R | 0.001 ± 0.004 | 0.003 ± 0.003 | 0.003 ± 0.003 | 0.9505 |  |  |  |
| A6cvl_L | 0.019 ± 0.007 | 0.004 ± 0.004 | 0.009 ± 0.004 | 0.1598 |  |  |  |
| A6cvl_R | 0.006 ± 0.005 | 0.011 ± 0.005 | 0.009 ± 0.003 | 0.7075 |  |  |  |
| A1_2_3ll_ R | 0.003 ± 0.004 | 0.009 ± 0.005 | 0.002 ± 0.002 | 0.3441 |  |  |  |
| A4ll_ R | 0.013 ± 0.005 | 0.013 ± 0.006 | 0.010 ± 0.003 | 0.8034 |  |  |  |
| A1_2_3ulhf_L | 0.017 ± 0.005 | 0.006 ± 0.003 | 0.015 ± 0.004 | 0.2903 |  |  |  |
| A1_2_3ulhf_R | 0.020 ± 0.007 | 0.015 ± 0.005 | 0.010 ± 0.003 | 0.3017 |  |  |  |
| A1_2_3tonIa_L | 0.008 ± 0.006 | 0.017 ± 0.006 | 0.008 ± 0.003 | 0.4577 |  |  |  |
| A1_2_3tonIa_R | 0.003 ± 0.004 | 0.012 ± 0.005 | 0.010 ± 0.003 | 0.3320 |  |  |  |
| A2_L | 0.013 ± 0.006 | 0.005 ± 0.003 | 0.014 ± 0.003 | 0.2799 |  |  |  |
| A2_R | 0.024 ± 0.009 | 0.009 ± 0.004 | 0.011 ± 0.004 | 0.1713 |  |  |  |
| A1_2_3tru_L | 0.017 ± 0.006 | 0.017 ± 0.007 | 0.016 ± 0.004 | 0.9822 |  |  |  |
| A1_2_3tru_R | 0.007 ± 0.004 | 0.013 ± 0.004 | 0.012 ± 0.004 | 0.5861 |  |  |  |
| A24cd_L | 0.008 ± 0.006 | 0.016 ± 0.006 | 0.013 ± 0.003 | 0.5705 |  |  |  |
| A24cd_R | 0.019 ± 0.005 | 0.019 ± 0.006 | 0.011 ± 0.004 | 0.4066 |  |  |  |
| A23c_L | 0.013 ± 0.005 | -0.003 ± 0.004 | 0.008 ± 0.003 | 0.0191 | 0.0185 | 0.8580 | 0.0977 |
| A23c_R | 0.005 ± 0.005 | 0.010 ± 0.004 | 0.010 ± 0.003 | 0.6717 |  |  |  |
| mPMtha_L | -0.004 ± 0.002 | 0.001 ± 0.003 | 0.002 ± 0.004 | 0.5289 |  |  |  |
| mPMtha_R | 0.011 ± 0.006 | 0.022 ± 0.007 | 0.012 ± 0.004 | 0.3302 |  |  |  |
| Stha_L | 0.007 ± 0.010 | 0.008 ± 0.005 | 0.003 ± 0.002 | 0.7180 |  |  |  |
| Stha_R | -0.004 ± 0.002 | 0.008 ± 0.004 | 0.001 ± 0.003 | 0.1029 |  |  |  |

*****Nodal vulnerability was calculated with one-way ANOVA test. The value in the table = mean value ± standard error mean. Post-hoc analysis with bonferroni correction was applied. SMA group = patients with supplementary motor area syndrome. Non-SMA group = patients without supplementary motor area syndrome. **Table S10. Nodal efficiency compared between the patients and healthy groups** **when glioma grew in the right hemisphere**

| Node name | SMA group | non-SMA group | Health group | One-way ANOVA  (*p* value) | SMA vs non-SMA  (*p* value) | SMA vs Health  (*p* value) | non-SMA vs Health  (*p* value) |
| --- | --- | --- | --- | --- | --- | --- | --- |
| A6dl_L^#^ | 0.265 ± 0.036 | 0.176 ± 0.011 | 0.168 ± 0.006 | 0.0006 | 0.1022 | 0.0594 | 0.8890 |
| A6m_ L^#^ | 0.247 ± 0.034 | 0.176 ± 0.007 | 0.171 ± 0.006 | 0.0042 | 0.0245 | 0.0044 | > 0.9999 |
| A6vl_ L^#^ | 0.269 ± 0.039 | 0.183 ± 0.008 | 0.170 ± 0.005 | 0.0006 | 0.1368 | 0.0723 | 0.4463 |
| A6cdl_ L^#^ | 0.230 ± 0.020 | 0.181 ± 0.009 | 0.171 ± 0.005 | 0.0007 | 0.0172 | 0.0006 | > 0.9999 |
| A4ul_L^#^ | 0.288 ± 0.037 | 0.175 ± 0.007 | 0.179 ± 0.004 | < 0.0001 | 0.0328 | 0.0380 | 0.9663 |
| A4ul_R^#^ | 0.232 ± 0.016 | 0.171 ± 0.007 | 0.169 ± 0.005 | < 0.0001 | 0.0001 | < 0.0001 | > 0.9999 |
| A4t_L^#^ | 0.283 ± 0.040 | 0.187 ± 0.010 | 0.175 ± 0.004 | 0.0003 | 0.1017 | 0.0554 | 0.6686 |
| A4t_R^#^ | 0.256 ± 0.021 | 0.171 ± 0.011 | 0.170 ± 0.005 | < 0.0001 | < 0.0001 | < 0.0001 | > 0.9999 |
| A4tl_L^#^ | 0.276 ± 0.046 | 0.170 ± 0.007 | 0.172 ± 0.006 | 0.0016 | 0.1216 | 0.1289 | 0.9964 |
| A4tl_R^#^ | 0.263 ± 0.043 | 0.167 ± 0.012 | 0.166 ± 0.006 | 0.0029 | 0.1480 | 0.1373 | > 0.9999 |
| A6cvl_L^#^ | 0.303 ± 0.046 | 0.182 ± 0.008 | 0.176 ± 0.005 | 0.0001 | 0.0647 | 0.0500 | 0.9259 |
| A6cvl_R^#^ | 0.223 ± 0.010 | 0.181 ± 0.009 | 0.182 ± 0.005 | 0.0003 | 0.0019 | 0.0004 | > 0.9999 |
| A1_2_3ll_ L^#^ | 0.293 ± 0.042 | 0.178 ± 0.013 | 0.171 ± 0.006 | 0.0002 | 0.0633 | 0.0422 | 0.9554 |
| A4ll_ L^#^ | 0.295 ± 0.049 | 0.177 ± 0.011 | 0.179 ± 0.006 | 0.0011 | 0.1012 | 0.1051 | 0.9972 |
| A1_2_3ulhf_L^#^ | 0.236 ± 0.012 | 0.197 ± 0.009 | 0.187 ± 0.006 | 0.0004 | 0.0157 | 0.0003 | > 0.9999 |
| A1_2_3ulhf_R^#^ | 0.234 ± 0.014 | 0.191 ± 0.011 | 0.185 ± 0.005 | 0.0012 | 0.0145 | 0.0011 | > 0.9999 |
| A1_2_3tonIa_L^#^ | 0.262 ± 0.035 | 0.182 ± 0.008 | 0.177 ± 0.005 | 0.0013 | 0.1296 | 0.0939 | 0.9401 |
| A1_2_3tonIa_R^#^ | 0.294 ± 0.045 | 0.185 ± 0.010 | 0.173 ± 0.005 | 0.0003 | 0.1046 | 0.0609 | 0.6943 |
| A2_L^#^ | 0.293 ± 0.049 | 0.176 ± 0.008 | 0.182 ± 0.004 | 0.0014 | 0.1109 | 0.1327 | 0.9124 |
| A2_R^#^ | 0.283 ± 0.041 | 0.179 ± 0.009 | 0.184 ± 0.006 | 0.0009 | 0.0848 | 0.1003 | 0.9705 |
| A1_2_3tru_L^#^ | 0.296 ± 0.043 | 0.183 ± 0.008 | 0.178 ± 0.006 | 0.0002 | 0.0671 | 0.0512 | 0.9400 |
| A1_2_3tru_R^#^ | 0.289 ± 0.043 | 0.168 ± 0.009 | 0.177 ± 0.005 | 0.0002 | 0.0468 | 0.0671 | 0.8009 |
| A24cd_L^#^ | 0.289 ± 0.042 | 0.192 ± 0.010 | 0.192 ± 0.007 | 0.0020 | 0.1264 | 0.1195 | > 0.9999 |
| A24cd_R^#^ | 0.259 ± 0.040 | 0.192 ± 0.009 | 0.191 ± 0.008 | 0.0338 | 0.3410 | .03274 | 0.9998 |
| A23c_L^#^ | 0.281 ± 0.042 | 0.183 ± 0.011 | 0.171 ± 0.006 | 0.0007 | 0.1266 | 0.0725 | 0.7281 |
| A23c_R^#^ | 0.243 ± 0.021 | 0.190 ± 0.010 | 0.179 ± 0.006 | 0.0008 | 0.0169 | 0.0006 | > 0.9999 |
| mPMtha_L^#^ | 0.254 ± 0.044 | 0.163 ± 0.007 | 0.156 ± 0.005 | 0.0027 | 0.1884 | 0.1419 | 0.8042 |
| mPMtha_R^#^ | 0.274 ± 0.046 | 0.181 ± 0.012 | 0.172 ± 0.005 | 0.0036 | 0.2150 | 0.1434 | 0.8556 |
| Stha_L^#^ | 0.235 ± 0.034 | 0.168 ± 0.008 | 0.165 ± 0.006 | 0.0093 | 0.2309 | 0.1936 | 0.9839 |
| Stha_R^#^ | 0.286 ± 0.048 | 0.170 ± 0.010 | 0.165 ± 0.006 | 0.0007 | 0.1035 | 0.0826 | 0.9673 |

* The nodal efficiency was calculated with one-way ANOVA test. The value in the table = mean value ± standard error mean. Post-hoc analysis with Tamhane correction (^#^) was applied. SMA group = patients with supplementary motor area syndrome. Non-SMA group = patients without supplementary motor area syndrome.

**Table S11. Nodal local efficiency compared between the patients and healthy groups** **when glioma grew in the right hemisphere**

| Node name | SMA group | non-SMA group | Health group | One-way ANOVA  (*p* value) | SMA vs non-SMA  (*p* value) | SMA vs Health  (*p* value) | non-SMA vs Health  (*p* value) |
| --- | --- | --- | --- | --- | --- | --- | --- |
| A6dl_L | 0.026 ± 0.006 | 0.037 ± 0.005 | 0.044 ± 0.004 | 0.0410 | 0.4813 | 0.0358 | 0.9848 |
| A6m_ L | 0.026 ± 0.006 | 0.050 ± 0.008 | 0.041 ± 0.004 | 0.0271 | 0.0237 | 0.1899 | 0.6235 |
| A6vl_ L | 0.026 ± 0.006 | 0.034 ± 0.004 | 0.039 ± 0.005 | 0.2021 | - | - | - |
| A6cdl_ L | 0.029 ± 0.007 | 0.038 ± 0.004 | 0.039 ± 0.004 | 0.3684 | - | - | - |
| A4ul_L | 0.026 ± 0.006 | 0.035 ± 0.005 | 0.042 ± 0.004 | 0.0998 | - | - | - |
| A4ul_R | 0.022 ± 0.005 | 0.039 ± 0.004 | 0.042 ± 0.003 | 0.0042 | 0.0384 | 0.0037 | > 0.9999 |
| A4t_L | 0.028 ± 0.006 | 0.048 ± 0.005 | 0.037 ± 0.003 | 0.0345 | 0.0299 | 0.5015 | 0.2971 |
| A4t_R | 0.023 ± 0.006 | 0.044 ± 0.007 | 0.041 ± 0.004 | 0.0235 | 0.0413 | 0.0456 | > 0.9999 |
| A4tl_L | 0.033 ± 0.008 | 0.041 ± 0.004 | 0.040 ± 0.004 | 0.5414 | - | - | - |
| A4tl_R | 0.024 ± 0.005 | 0.035 ± 0.003 | 0.041 ± 0.004 | 0.0337 | 0.4381 | 0.0287 | 0.9493 |
| A6cvl_L | 0.022 ± 0.005 | 0.039 ± 0.003 | 0.041 ± 0.004 | 0.0050 | 0.0295 | 0.0053 | > 0.9999 |
| A6cvl_R | 0.033 ± 0.011 | 0.044 ± 0.007 | 0.041 ± 0.003 | 0.5512 | - | - | - |
| A1_2_3ll_ L | 0.031 ± 0.006 | 0.048 ± 0.007 | 0.049 ± 0.004 | 0.0660 | - | - | - |
| A4ll_ L | 0.027 ± 0.006 | 0.049 ± 0.010 | 0.042 ± 0.004 | 0.0988 | - | - | - |
| A1_2_3ulhf_L | 0.025 ± 0.005 | 0.030 ± 0.004 | 0.042 ± 0.004 | 0.0235 | > 0.9999 | 0.0307 | 0.2274 |
| A1_2_3ulhf_R | 0.027 ± 0.006 | 0.037 ± 0.005 | 0.039 ± 0.004 | 0.1951 | - | - | - |
| A1_2_3tonIa_L | 0.024 ± 0.005 | 0.041 ± 0.005 | 0.044 ± 0.003 | 0.0070 | 0.0523 | 0.0063 | > 0.9999 |
| A1_2_3tonIa_R | 0.025 ± 0.007 | 0.034 ± 0.004 | 0.049 ± 0.005 | 0.0112 | 0.9895 | 0.0129 | 0.1958 |
| A2_L | 0.029 ± 0.008 | 0.037 ± 0.005 | 0.034 ± 0.003 | 0.6060 | - | - | - |
| A2_R | 0.022 ± 0.005 | 0.038 ± 0.005 | 0.035 ± 0.004 | 0.0672 | - | - | - |
| A1_2_3tru_L | 0.025 ± 0.005 | 0.032 ± 0.004 | 0.044 ± 0.004 | 0.0083 | 0.9718 | 0.0098 | 0.1635 |
| A1_2_3tru_R | 0.023 ± 0.005 | 0.042 ± 0.003 | 0.042 ± 0.003 | 0.0029 | 0.0116 | 0.0040 | > 0.9999 |
| A24cd_L | 0.028 ± 0.006 | 0.048 ± 0.007 | 0.035 ± 0.003 | 0.0406 | 0.0406 | 0.8773 | 0.1938 |
| A24cd_R | 0.021 ± 0.005 | 0.041 ± 0.004 | 0.038 ± 0.004 | 0.0108 | 0.0181 | 0.0255 | > 0.9999 |
| A23c_L | 0.026 ± 0.007 | 0.046 ± 0.006 | 0.040 ± 0.003 | 0.0368 | 0.0387 | 0.1415 | > 0.9999 |
| A23c_R | 0.017 ± 0.003 | 0.035 ± 0.004 | 0.036 ± 0.004 | 0.0041 | 0.0224 | 0.0045 | > 0.9999 |
| mPMtha_L | 0.024 ± 0.005 | 0.046 ± 0.006 | 0.036 ± 0.004 | 0.0140 | 0.0112 | 0.1503 | 0.4353 |
| mPMtha_R | 0.025 ± 0.006 | 0.031 ± 0.003 | 0.041 ± 0.003 | 0.0258 | > 0.9999 | 0.0306 | 0.2864 |
| Stha_L | 0.032 ± 0.010 | 0.047 ± 0.009 | 0.036 ± 0.003 | 0.3640 | - | - | - |
| Stha_R | 0.025 ± 0.005 | 0.035 ± 0.004 | 0.036 ± 0.004 | 0.1906 | - | - | - |

***** Nodal local efficiency was calculated with one-way ANOVA test. The value in the table = mean value ± standard error mean. Post-hoc analysis with bonferroni correction was applied. SMA group = patients with supplementary motor area syndrome. Non-SMA group = patients without supplementary motor area syndrome.

**Table S12. Degree centrality compared between the patients and healthy groups** **when glioma grew in the right hemisphere**

| Node name | SMA group | non-SMA group | Health group | One-way ANOVA  (*p* value) | SMA vs non-SMA  (*p* value) | SMA vs Health  (*p* value) | non-SMA vs Health  (*p* value) |
| --- | --- | --- | --- | --- | --- | --- | --- |
| A6dl_L^#^ | 3.305 ± 0.576 | 2.042 ± 0.199 | 1.826 ± 0.104 | 0.0016 | 0.1684 | 0.0771 | 0.7351 |
| A6m_ L^#^ | 2.858 ± 0.498 | 1.908 ± 0.127 | 1.932 ± 0.111 | 0.0214 | 0.2490 | 0.2637 | 0.9987 |
| A6vl_ L^#^ | 3.309 ± 0.538 | 2.013 ± 0.146 | 1.783 ± 0.092 | 0.0003 | 0.1088 | 0.0460 | 0.4942 |
| A6cdl_ L^#^ | 2.883 ± 0.452 | 2.129 ± 0.164 | 1.922 ± 0.091 | 0.0136 | 0.3744 | 0.1698 | 0.6491 |
| A4ul_L^#^ | 3.905 ± 0.639 | 1.959 ± 0.149 | 2.124 ± 0.080 | 0.0001 | 0.0319 | 0.0497 | 0.7272 |
| A4ul_R^#^ | 2.474 ± 0.193 | 1.830 ± 0.147 | 1.821 ± 0.101 | 0.0031 | 0.0150 | 0.0038 | > 0.9999 |
| A4t_L^#^ | 3.642 ± 0.651 | 2.282 ± 0.141 | 1.890 ± 0.079 | 0.0005 | 0.1830 | 0.0597 | 0.0756 |
| A4t_R^#^ | 2.583 ± 0.193 | 1.787 ± 0.184 | 1.877 ± 0.094 | 0.0011 | 0.0029 | 0.0025 | > 0.9999 |
| A4tl_L^#^ | 3.485 ± 0.770 | 1.712 ± 0.127 | 1.953 ± 0.107 | 0.0041 | 0.1237 | 0.2084 | 0.4183 |
| A4tl_R^#^ | 2.938 ± 0.618 | 1.710 ± 0.181 | 1.717 ± 0.086 | 0.0100 | 0.2258 | 0.2130 | > 0.9999 |
| A6cvl_L^#^ | 3.830 ± 0.708 | 2.094 ± 0.123 | 2.024 ± 0.121 | 0.0007 | 0.0950 | 0.0790 | 0.9711 |
| A6cvl_R^#^ | 3.747 ± 0.688 | 1.987 ± 0.155 | 2.050 ± 0.084 | 0.0006 | 0.0803 | 0.0904 | 0.9805 |
| A1_2_3ll_ L^#^ | 4.029 ± 0.682 | 2.103 ± 0.265 | 1.894 ± 0.125 | 0.0001 | 0.0568 | 0.0261 | 0.8720 |
| A4ll_ L^#^ | 3.842 ± 0.803 | 2.035 ± 0.228 | 2.109 ± 0.113 | 0.0038 | 0.1450 | 0.1570 | 0.9894 |
| A1_2_3ulhf_L^#^ | 3.403 ± 0.491 | 2.409 ± 0.146 | 2.131 ± 0.099 | 0.0017 | 0.2136 | 0.0745 | 0.3531 |
| A1_2_3ulhf_R^#^ | 3.257 ± 0.570 | 2.206 ± 0.212 | 2.149 ± 0.102 | 0.0190 | 0.2965 | 0.2277 | 0.9937 |
| A1_2_3tonIa_L^#^ | 3.189 ± 0.522 | 2.065 ± 0.148 | 2.013 ± 0.101 | 0.0046 | 0.1718 | 0.1367 | 0.9889 |
| A1_2_3tonIa_R^#^ | 3.630 ± 0.650 | 2.097 ± 0.188 | 1.969 ± 0.111 | 0.0011 | 0.1206 | 0.0780 | 0.9216 |
| A2_L^#^ | 3.934 ± 0.838 | 1.983 ± 0.191 | 2.037 ± 0.084 | 0.0017 | 0.1223 | 0.1296 | 0.9926 |
| A2_R^#^ | 3.744 ± 0.731 | 2.010 ± 0.164 | 2.119 ± 0.136 | 0.0029 | 0.1128 | 0.1434 | 0.9452 |
| A1_2_3tru_L^#^ | 3.788 ± 0.693 | 2.036 ± 0.123 | 2.066 ± 0.115 | 0.0007 | 0.0826 | 0.0889 | 0.9974 |
| A1_2_3tru_R^#^ | 3.613 ± 0.657 | 1.854 ± 0.156 | 2.041 ± 0.088 | 0.0007 | 0.0648 | 0.1037 | 0.6827 |
| A24cd_L^#^ | 3.653 ± 0.591 | 2.251 ± 0.167 | 2.317 ± 0.118 | 0.0033 | 0.1175 | 0.1354 | 0.9852 |
| A24cd_R^#^ | 3.321 ± 0.699 | 2.276 ± 0.215 | 2.223 ± 0.164 | 0.0778 | - | - | - |
| A23c_L^#^ | 3.644 ± 0.745 | 2.070 ± 0.199 | 1.925 ± 0.094 | 0.0026 | 0.1813 | 0.1209 | 0.8948 |
| A23c_R^#^ | 2.850 ± 0.188 | 2.212 ± 0.175 | 2.033 ± 0.128 | 0.0022 | 0.0462 | 0.0016 | > 0.9999 |
| mPMtha_L^#^ | 2.993 ± 0.721 | 1.578 ± 0.118 | 1.561 ± 0.087 | 0.0072 | 0.2195 | 0.2085 | 0.9993 |
| mPMtha_R^#^ | 3.572 ± 0.811 | 2.140 ± 0.240 | 2.004 ± 0.095 | 0.0141 | 0.3160 | 0.2271 | 0.9428 |
| Stha_L^#^ | 2.837 ± 0.538 | 1.735 ± 0.154 | 1.745 ± 0.117 | 0.0118 | 0.2044 | 0.2019 | > 0.9999 |
| Stha_R^#^ | 3.853 ± 0.879 | 1.759 ± 0.159 | 1.792 ± 0.111 | 0.0012 | 0.1080 | 0.1129 | 0.9977 |

*****Degree centrality was calculated with one-way ANOVA test. The value in the table = mean value ± standard error mean. Post-hoc analysis with Tamhane correction (^#^) was applied. SMA group = patients with supplementary motor area syndrome. Non-SMA group = patients without supplementary motor area syndrome.

**Table S13. Nodal vulnerability compared between the patients and healthy groups** **when glioma grew in the right hemisphere**

| Node name | SMA group | non-SMA group | Health group | One-way ANOVA  (*p* value) | SMA vs non-SMA  (*p* value) | SMA vs Health  (*p* value) | non-SMA vs Health  (*p* value) |
| --- | --- | --- | --- | --- | --- | --- | --- |
| A6dl_L | 0.016 ± 0.006 | 0.004 ± 0.004 | 0.008 ± 0.003 | 0.2126 | - | - | - |
| A6m_ L | 0.006 ± 0.005 | 0.008 ± 0.004 | 0.007 ± 0.003 | 0.9722 | - | - | - |
| A6vl_ L | 0.007 ± 0.004 | 0.007 ± 0.004 | 0.003 ± 0.002 | 0.6357 | - | - | - |
| A6cdl_ L | 0.004 ± 0.004 | 0.005 ± 0.005 | 0.004 ± 0.003 | 0.9925 | - | - | - |
| A4ul_L | 0.032 ± 0.007 | 0.008 ± 0.004 | 0.010 ± 0.003 | 0.0009 | - | - | - |
| A4ul_R | 0.020 ± 0.007 | -0.001 ± 0.002 | 0.009 ± 0.004 | 0.0255 | 0.0209 | 0.3072 | 0.3690 |
| A4t_L | 0.003 ± 0.004 | 0.010 ± 0.003 | 0.013 ± 0.004 | 0.1842 | - | - | - |
| A4t_R | 0.009 ± 0.006 | 0.002 ± 0.005 | 0.012 ± 0.004 | 0.3975 | - | - | - |
| A4tl_L | 0.015 ± 0.006 | 0.006 ± 0.007 | 0.007 ± 0.003 | 0.4971 | - | - | - |
| A4tl_R | -0.004 ± 0.002 | 0.002 ± 0.003 | 0.006 ± 0.003 | 0.0892 | - | - | - |
| A6cvl_L | 0.010 ± 0.005 | 0.013 ± 0.004 | 0.014 ± 0.006 | 0.8584 | - | - | - |
| A6cvl_R | 0.007 ± 0.004 | 0.012 ± 0.005 | 0.012 ± 0.003 | 0.6770 | - | - | - |
| A1_2_3ll_ L | 0.012 ± 0.006 | 0.006 ± 0.006 | 0.011 ± 0.006 | 0.8125 | - | - | - |
| A4ll_ L | 0.014 ± 0.006 | 0.014 ± 0.008 | 0.019 ± 0.005 | 0.7876 | - | - | - |
| A1_2_3ulhf_L | 0.018 ± 0.004 | 0.020 ± 0.005 | 0.010 ± 0.003 | 0.0920 | - | - | - |
| A1_2_3ulhf_R | 0.006 ± 0.004 | 0.016 ± 0.005 | 0.013 ± 0.004 | 0.4675 | - | - | - |
| A1_2_3tonIa_L | 0.005 ± 0.005 | 0.014 ± 0.006 | 0.008 ± 0.003 | 0.4946 | - | - | - |
| A1_2_3tonIa_R | 0.010 ± 0.007 | 0.012 ± 0.005 | 0.005 ± 0.002 | 0.4245 | - | - | - |
| A2_L | 0.013 ± 0.004 | 0.008 ± 0.005 | 0.011 ± 0.003 | 0.6473 | - | - | - |
| A2_R | 0.014 ± 0.006 | 0.009 ± 0.004 | 0.024 ± 0.007 | 0.2277 | - | - | - |
| A1_2_3tru_L | 0.014 ± 0.005 | 0.009 ± 0.004 | 0.012 ± 0.004 | 0.8103 | - | - | - |
| A1_2_3tru_R | 0.012 ± 0.006 | 0.002 ± 0.003 | 0.011 ± 0.003 | 0.2880 | - | - | - |
| A24cd_L | 0.013 ± 0.005 | 0.014 ± 0.006 | 0.022 ± 0.005 | 0.3647 | - | - | - |
| A24cd_R | 0.019 ± 0.009 | 0.022 ± 0.007 | 0.013 ± 0.005 | 0.5677 | - | - | - |
| A23c_L | 0.012 ± 0.004 | 0.009 ± 0.005 | 0.006 ± 0.004 | 0.6635 | - | - | - |
| A23c_R | 0.021 ± 0.007 | 0.003 ± 0.003 | 0.005 ± 0.002 | 0.0044 | 0.0085 | 0.0108 | > 0.9999 |
| mPMtha_L | 0.001 ± 0.004 | 0.002 ± 0.004 | 0.001 ± 0.003 | 0.9640 | - | - | - |
| mPMtha_R | 0.014 ± 0.009 | 0.012 ± 0.007 | 0.012 ± 0.004 | 0.9513 | - | - | - |
| Stha_L | -0.002 ± 0.004 | 0.004 ± 0.004 | 0.006 ± 0.004 | 0.3612 | - | - | - |
| Stha_R | 0.010 ± 0.006 | 0.002 ± 0.004 | 0.009 ± 0.005 | 0.5749 | - | - | - |

*****Nodal vulnerability was calculated with one-way ANOVA test. The value in the table = mean value ± standard error mean. Post-hoc analysis with bonferroni correction was applied. SMA group = patients with supplementary motor area syndrome. Non-SMA group = patients without supplementary motor area syndrome.

**Table S14. Testing the mediation effect of nodal efficiency of A4ul in the lesioned hemisphere on recovery time of patients with SMA syndrome**

|  | Effect | SE or Boot SE | t value | p value | Lower limited 95% CI | Upper limited 95% CI | Percentage of effect |
| --- | --- | --- | --- | --- | --- | --- | --- |
| Correlation between nodal efficiency of A4ul and surgical region or edema adjacent to A4ul | | | | | | | |
| Constant | 0.20 | 0.01 | 19.92 | < 0.0001 | 0.18 | 0.22 | - |
| Surgical region or edema adjacent to A4ul | 0.59 | 0.02 | 3.22 | 0.0030 | 0.02 | 0.10 | - |
| Indirect effect model | | | | | | | |
| Constant | -7.79 | 8.13 | -0.96 | 0.3461 | -24.40 | 8.82 | - |
| Surgical region or edema adjacent to A4ul | 15.71 | 4.60 | 3.42 | 0.0018 | 6.32 | 25.09 | - |
| Nodal efficiency of A4ul | 94.31 | 39.05 | 2.42 | 0.0220 | 14.55 | 174.07 | - |
| Total effect model | | | | | | | |
| Constant | 11.13 | 2.35 | 4.73 | < 0.0001 | 6.33 | 15.93 | - |
| Surgical region or edema adjacent to A4ul | 21.27 | 4.28 | 4.98 | < 0.0001 | 12.55 | 29.99 | - |
| Summary | | | | | | | |
| Total effect | 21.27 | 4.28 | 4.98 | < 0.0001 | 12.55 | 29.99 | - |
| Direct effect | 15.71 | 4.60 | 3.42 | 0.0018 | 6.32 | 25.09 | 73.86% |
| Indirect effect | 5.56 | 3.48 |  | 0.0220 | 0.84 | 12.39 | 26.14% |

***** A4ul = Upper limb region of Brodmann area 4, CI = confident interval.

**Table S15. Testing the mediation effect of nodal vulnerability of A4ul in the lesioned hemisphere on recovery time of patients with SMA syndrome**

|  | Effect | SE or Boot SE | t value | p value | Lower limited 95% CI | Upper limited 95% CI | Percentage of effect |
| --- | --- | --- | --- | --- | --- | --- | --- |
| Correlation between nodal vulnerability of A4ul and surgical region or edema involved in A4ul | | | | | | |  |
| Constant | 0.01 | 0.01 | 0.78 | 0.4434 | -0.01 | 0.21 | - |
| Surgical region or edema involved in A4ul | 0.05 | 0.01 | 3.47 | 0.0016 | 0.02 | 0.07 | - |
| Indirect effect model | | | | | | | |
| Constant | 9.75 | 1.58 | 6.17 | < 0.0001 | 6.52 | 12.98 | - |
| Surgical region or edema involved in A4ul | 10.07 | 3.35 | 3.00 | 0.0053 | 3.22 | 16.91 | - |
| Nodal vulnerability of A4ul | 243.12 | 38.43 | 6.33 | < 0.0001 | 164.64 | 321.60 | - |
| Total effect model | | | | | | | |
| Constant | 11.13 | 2.35 | 4.73 | < 0.0001 | 6.33 | 15.93 | - |
| Surgical region or edema involved in A4ul | 21.27 | 4.28 | 4.98 | < 0.0001 | 12.55 | 29.99 | - |
| Summary | | | | | | | |
| Total effect | 21.27 | 4.28 | 4.98 | < 0.0001 | 12.55 | 29.99 | - |
| Direct effect | 10.07 | 3.35 | 3.00 | 0.0053 | 3.22 | 16.91 | 47.34% |
| Indirect effect | 11.20 | 5.00 |  | < 0.0001 | 1.81 | 21.73 | 52.66% |

***** A4ul = Upper limb region of Brodmann area 4, CI = confident interval.

**Table S16. Testing the mediation effect of nodal efficiency of A23c in the lesioned hemisphere on recovery time of patients with SMA syndrome**

|  | Effect | SE or Boot SE | t value | p value | Lower limited 95% CI | Upper limited 95% CI | Percentage of effect |
| --- | --- | --- | --- | --- | --- | --- | --- |
| Correlation between nodal efficiency of A23c and surgical region or edema involved in A23c | | | | | | | |
| Constant | 0.19 | 0.02 | 10.62 | < 0.0001 | 0.15 | 0.22 | - |
| Surgical region or edema involved in A23c | 0.05 | 0.02 | 2.28 | 0.0297 | 0.01 | 0.10 | - |
| Indirect effect model | | | | | | | |
| Constant | -6.21 | 6.54 | -0.95 | 0.3500 | -19.56 | 7.15 | - |
| Surgical region or edema involved in A23c | 11.16 | 4.44 | 2.51 | 0.0176 | 2.09 | 20.23 | - |
| Nodal efficiency of A23c | 81.54 | 30.91 | 2.64 | 0.0131 | 18.41 | 144.66 | - |
| Total effect model | | | | | | | |
| Constant | 9.07 | 3.32 | 2.73 | 0.0102 | 2.30 | 15.83 | - |
| Surgical region or edema involved in A23c | 15.60 | 4.49 | 3.48 | 0.0015 | 6.44 | 24.76 | - |
| Summary | | | | | | | |
| Total effect | 15.60 | 4.49 | 3.48 | 0.0015 | 6.44 | 24.76 | - |
| Direct effect | 11.16 | 4.44 | 2.51 | 0.0176 | 2.09 | 20.23 | 71.54% |
| Indirect effect | 4.44 | 3.37 |  | 0.0131 | 0.05 | 13.01 | 28.46% |

***** A23c = caudal area of Brodmann area 23, CI = confident interval.

**Table S17. Testing the mediation effect of nodal vulnerability of A23c in the lesioned hemisphere on recovery time of patients with SMA syndrome**

|  | Effect | SE or Boot SE | t value | p value | Lower limited 95% CI | Upper limited 95% CI | Percentage of effect |
| --- | --- | --- | --- | --- | --- | --- | --- |
| Correlation between nodal vulnerability of A23c and surgical region or edema involved in A23c | | | | | | |  |
| Constant | -0.01 | 0.01 | -0.26 | 0.7967 | -0.01 | 0.01 | - |
| Surgical region or edema involved in A23c | 0.02 | 0.01 | 2.40 | 0.0226 | 0.01 | 0.03 | - |
| Indirect effect model | | | | | | | |
| Constant | 9.52 | 2.88 | 3.30 | 0.0025 | 3.63 | 15.40 | - |
| Surgical region or edema involved in A23c | 9.99 | 4.24 | 2.36 | 0.0252 | 1.33 | 18.66 | - |
| Nodal vulnerability of A23c | 330.42 | 98.97 | 3.34 | 0.0023 | 128.28 | 532.56 | - |
| Total effect model | | | | | | | |
| Constant | 9.07 | 3.32 | 2.73 | 0.0102 | 2.30 | 15.83 | - |
| Surgical region or edema involved in A23c | 15.60 | 4.49 | 3.48 | 0.0015 | 6.44 | 24.76 | - |
| Summary | | | | | | | |
| Total effect | 15.60 | 4.49 | 3.48 | 0.0015 | 6.44 | 24.76 | - |
| Direct effect | 9.99 | 4.24 | 2.36 | 0.0252 | 1.33 | 18.66 | 64.04% |
| Indirect effect | 5.61 | 3.07 |  | 0.0023 | 0.54 | 12.39 | 35.96% |

***** A23c = caudal area of Brodmann area 23, CI = confident interval.

#
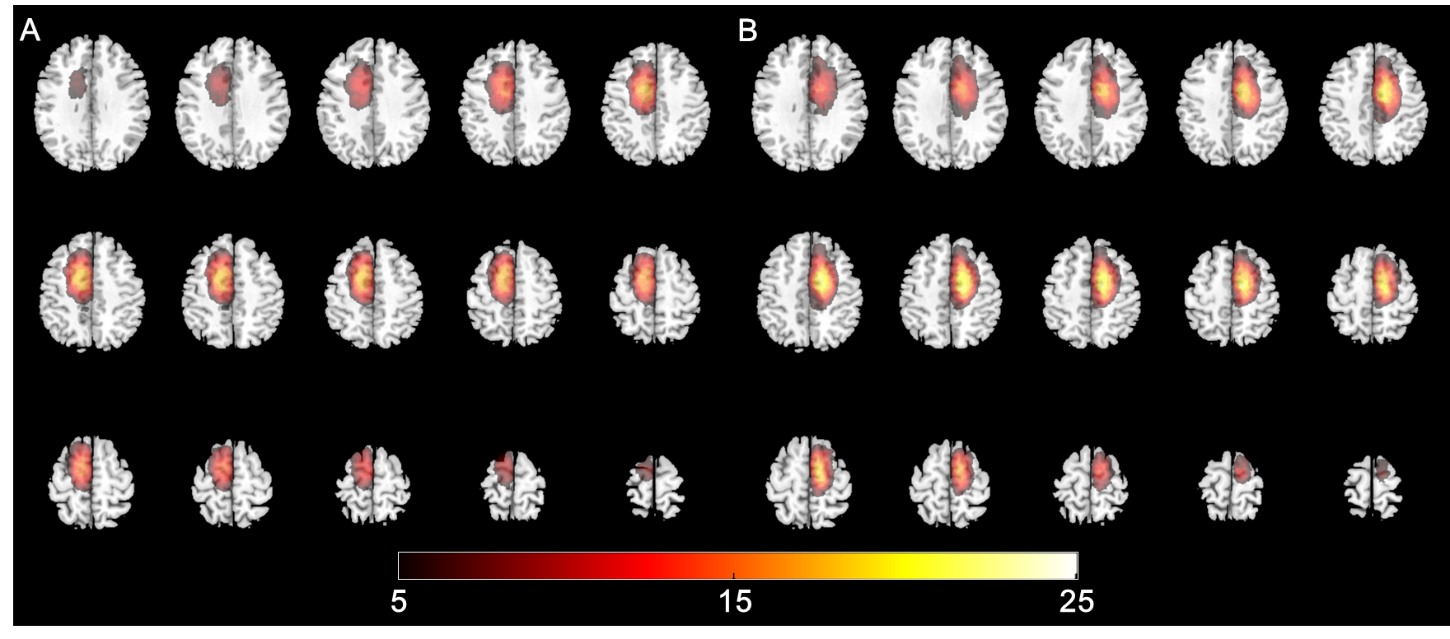
Part 3. Supplemental Figures

**Figure S1.** Tumor locations in patient with SMA glioma. A) Left hemispheric glioma; B) Right hemispheric glioma.


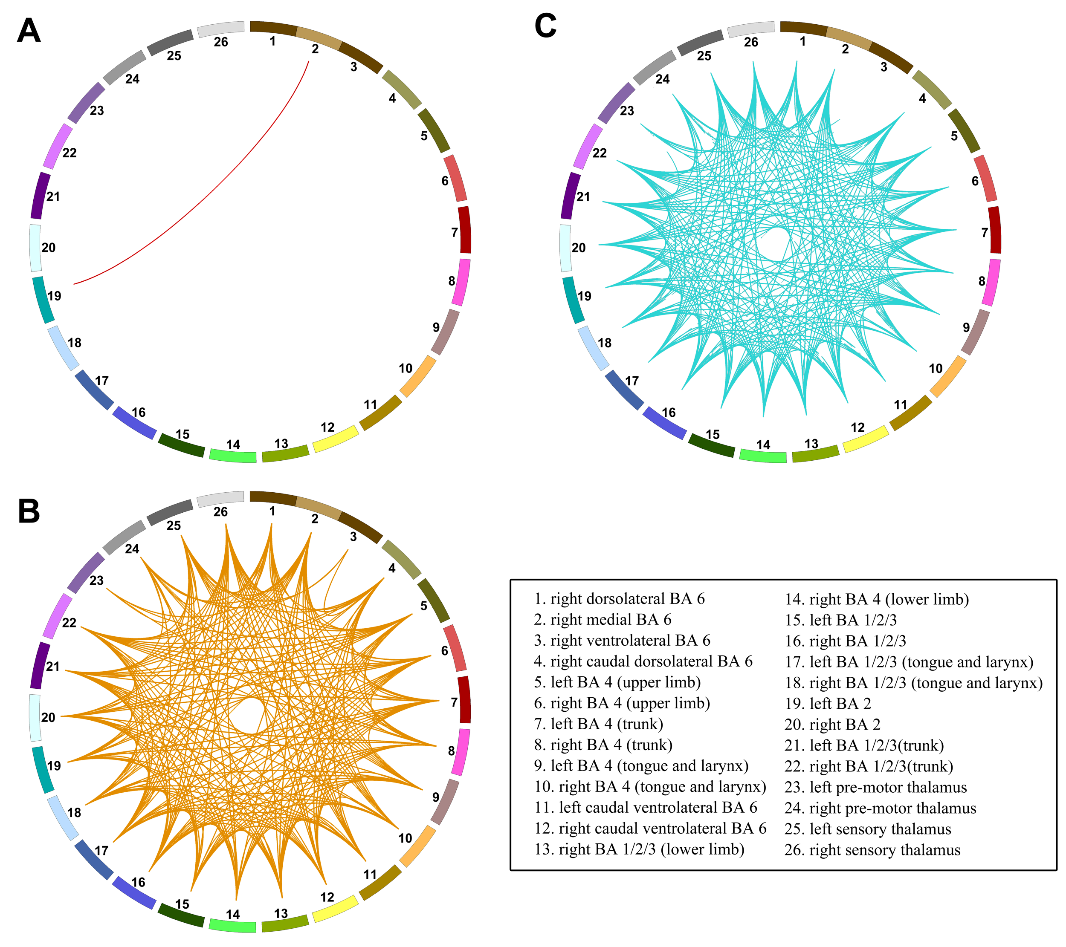


**Figure S2.** Significant alterations of the functional connectivity when respectively compared with each two groups for the left hemispheric gliomas. A) SMA-group vs non-SMA group. B) SMA-group vs healthy group. C) non-SMA group vs healthy group.


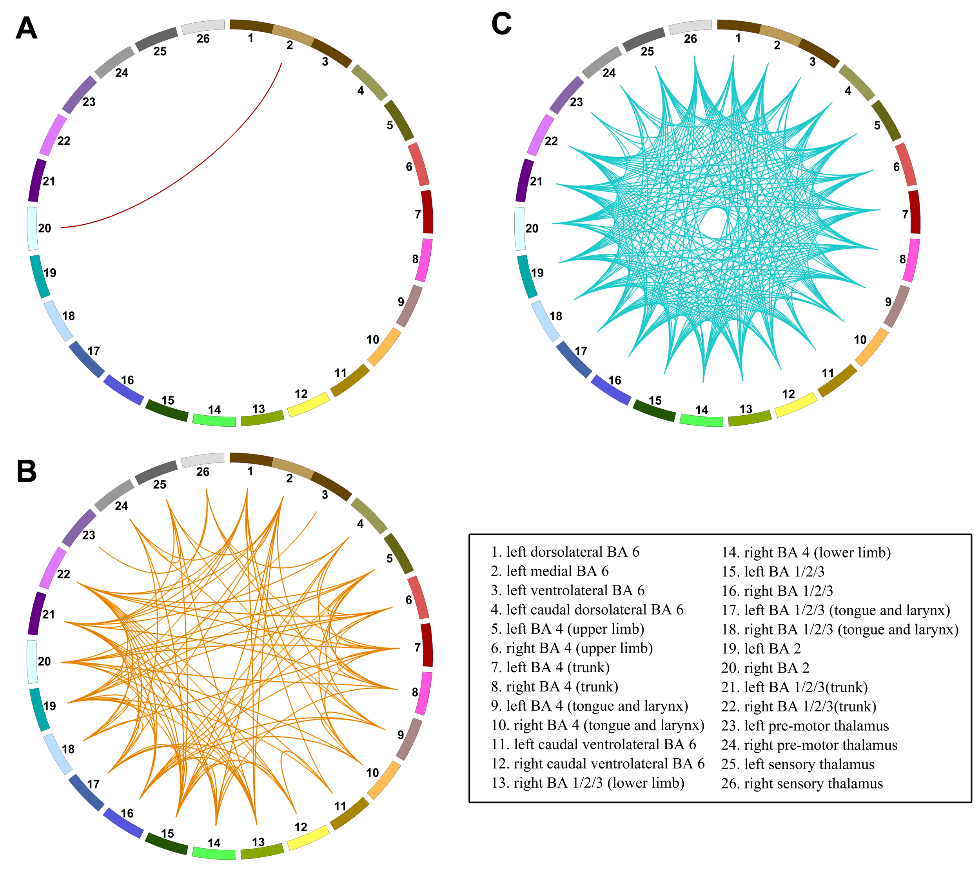


**Figure S3.** Significant alterations of the functional connectivity when respectively compared with each two groups for the right hemispheric gliomas. A) SMA-group vs non-SMA group. B) SMA-group vs healthy group. C) non-SMA group vs healthy group.
